# Supplementary material for: Novel Tough and Transparent Ultra-Extensible Nanocomposite Elastomers Based on Poly(2-methoxyethylacrylate) and Their Switching between Plasto-Elasticity and Viscoelasticity
Source: Polymers (Basel). 2021 Dec 4;13(23):4254. doi: 10.3390/polym13234254 (PMC8659642; doi:10.3390/polym13234254)
Supplement: Supplementary file 1 [file polymers-13-04254-s001.zip › polymers-1474548-supplementary.pdf]

# SUPPLEMENTARY INFORMATION

## Novel Tough and Transparent Ultra-Extensible Nanocomposite Elastomers Based on poly(2-methoxyethylacrylate) and Their Switching between Plasto-Elasticity and Viscoelasticity

Katarzyna Byś[1], Beata Strachota[1], Adam Strachota[1]*, Ewa Pavlova[1], Miloš Steinhart[1], Beata Mossety-Leszczak[2], Weronika Zając[3]

[1)] *Institute of Macromolecular Chemistry, Czech Academy of Sciences, Heyrovskeho nam. 2, CZ-162 06 Praha, Czech Republic*

[2)] *Rzeszow University of Technology, al. Powstancow Warszawy 6, 35-959 Rzeszow, Poland*

[3)] *Doctoral School of Engineering and Technical Sciences at the Rzeszow University of Technology, al. Powstancow Warszawy 12, 35-959 Rzeszow, Poland*

# 1. Synthesis

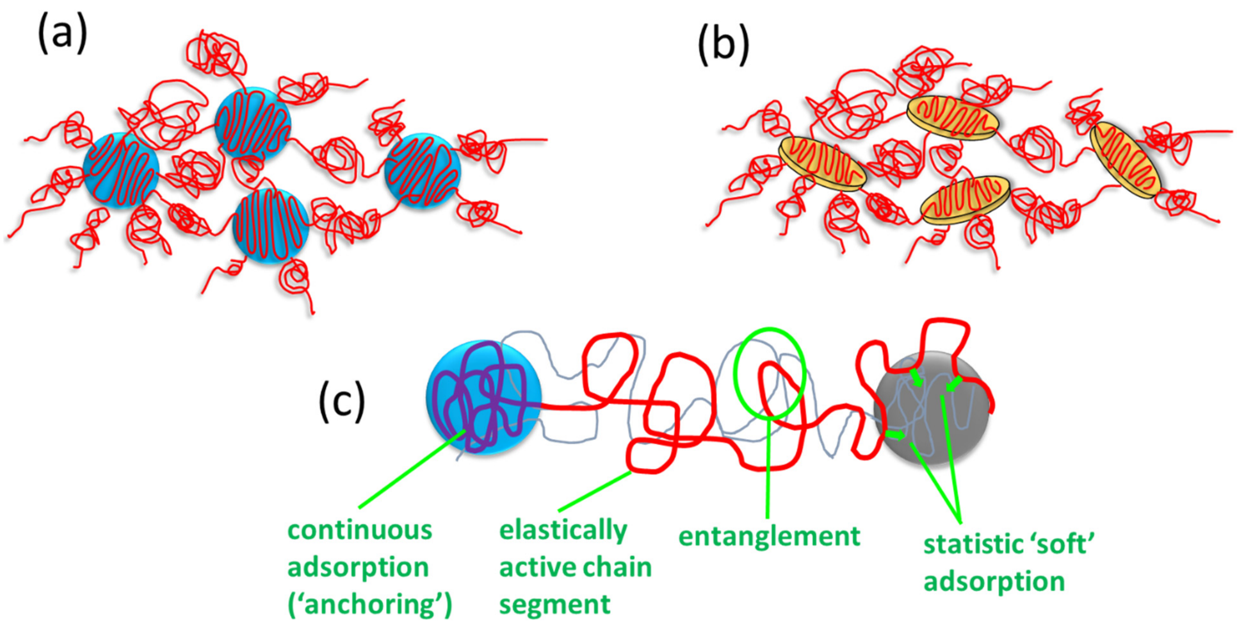


***SI-Figure S1.*** *Idealized self-assembly of the nanocomposite elastomeric networks polyMEA/nano-SiO2 (a) and polyMEA/clay (b), based entirely on physical crosslinking.*

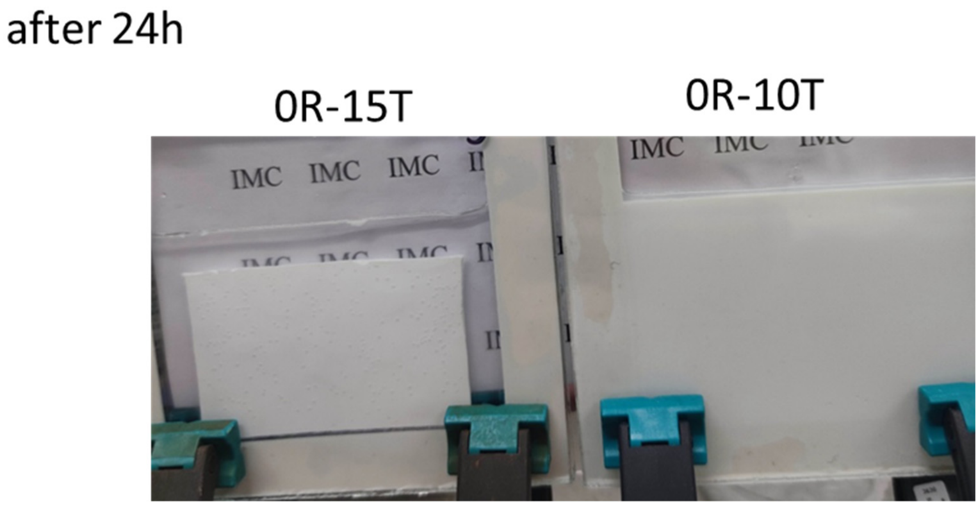


***SI- Figure S2.*** *Comparison of appearance of shrinking synthesis mixture with 15 or more wt.% of in-situ nano-silica (example: 0R-15T, left) with a non-shrinking one (example: 0R-10T, right); appearance after 24 h since the start of the synthesis.*

## Morphology: TEM ('as prepared' state): High-resolution images

***SI-Figure S2 – multi-page:***

# neat polyMEA

**(a)**

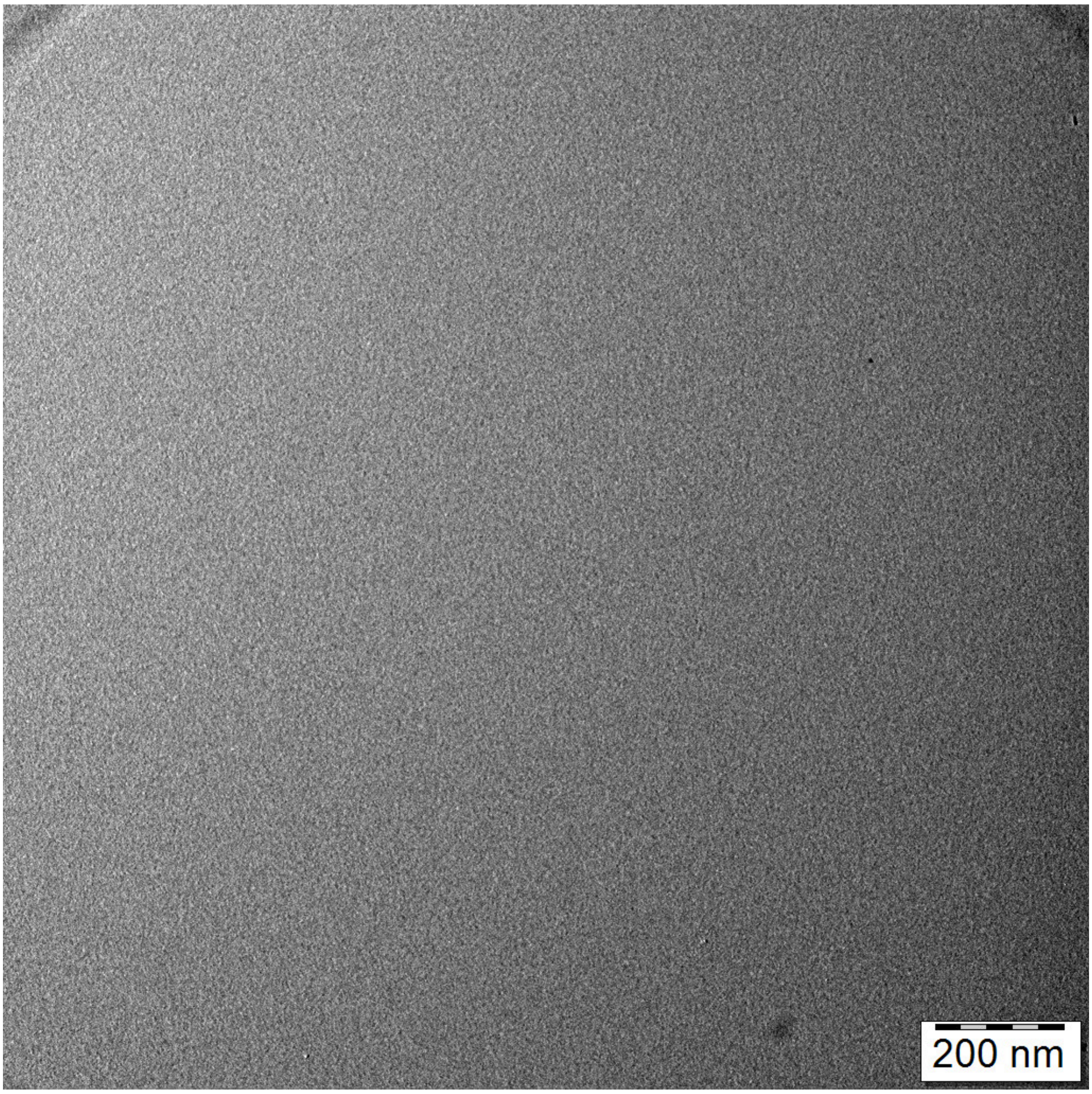


***SI-Figure S2 – multi-page:*** *(a) High-resolution version of the TEM image of neat polyMEA.*

***SI-Figure S2 – multi-page (continued):***

# 0R-5T

**(b)**

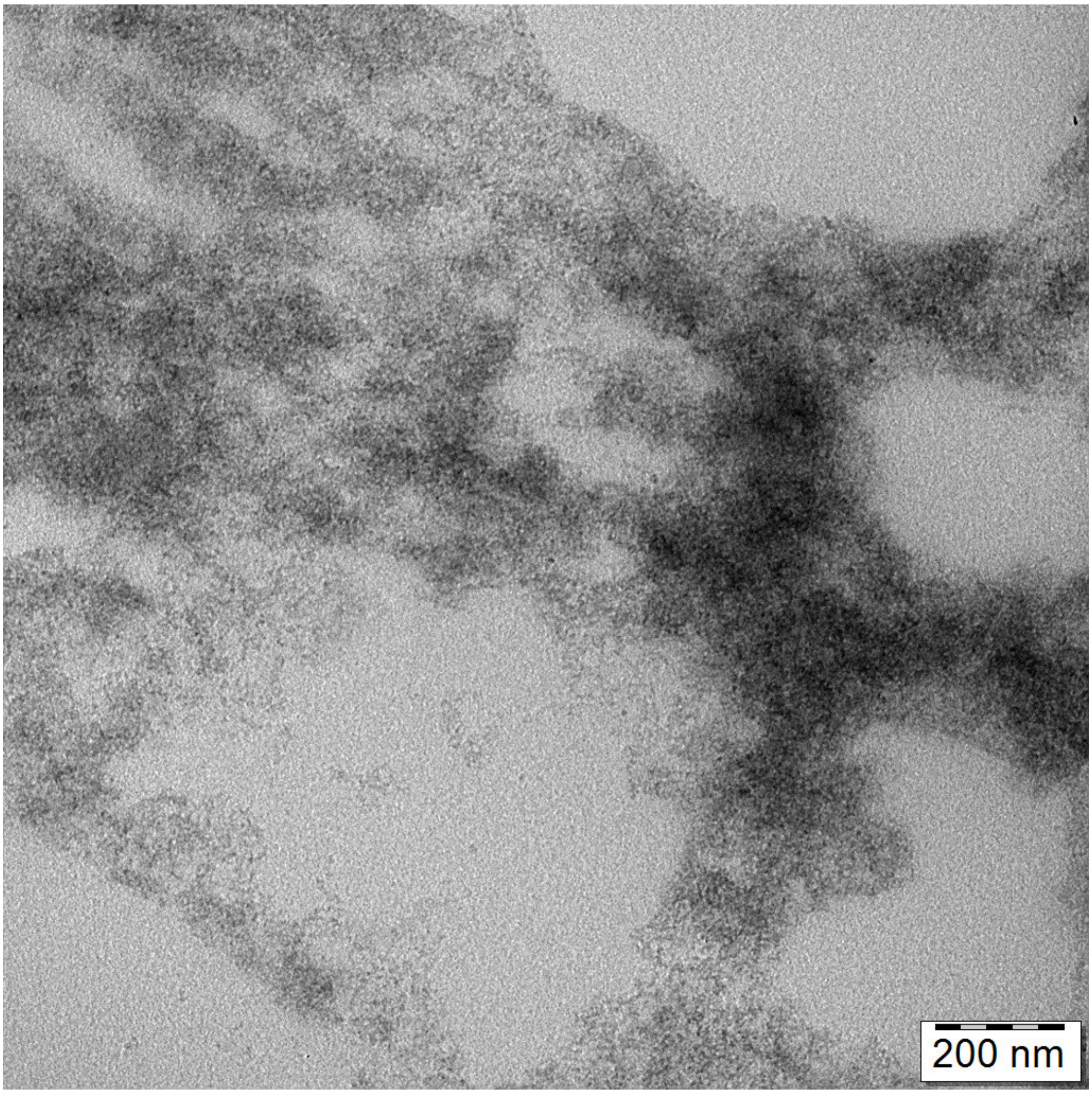


***SI-Figure S2 – multi-page (continued):*** *(b) High-resolution version of the TEM image of 0R-5T.*

***SI-Figure S2 – multi-page (continued):***

# 0R-15T

**(c)**

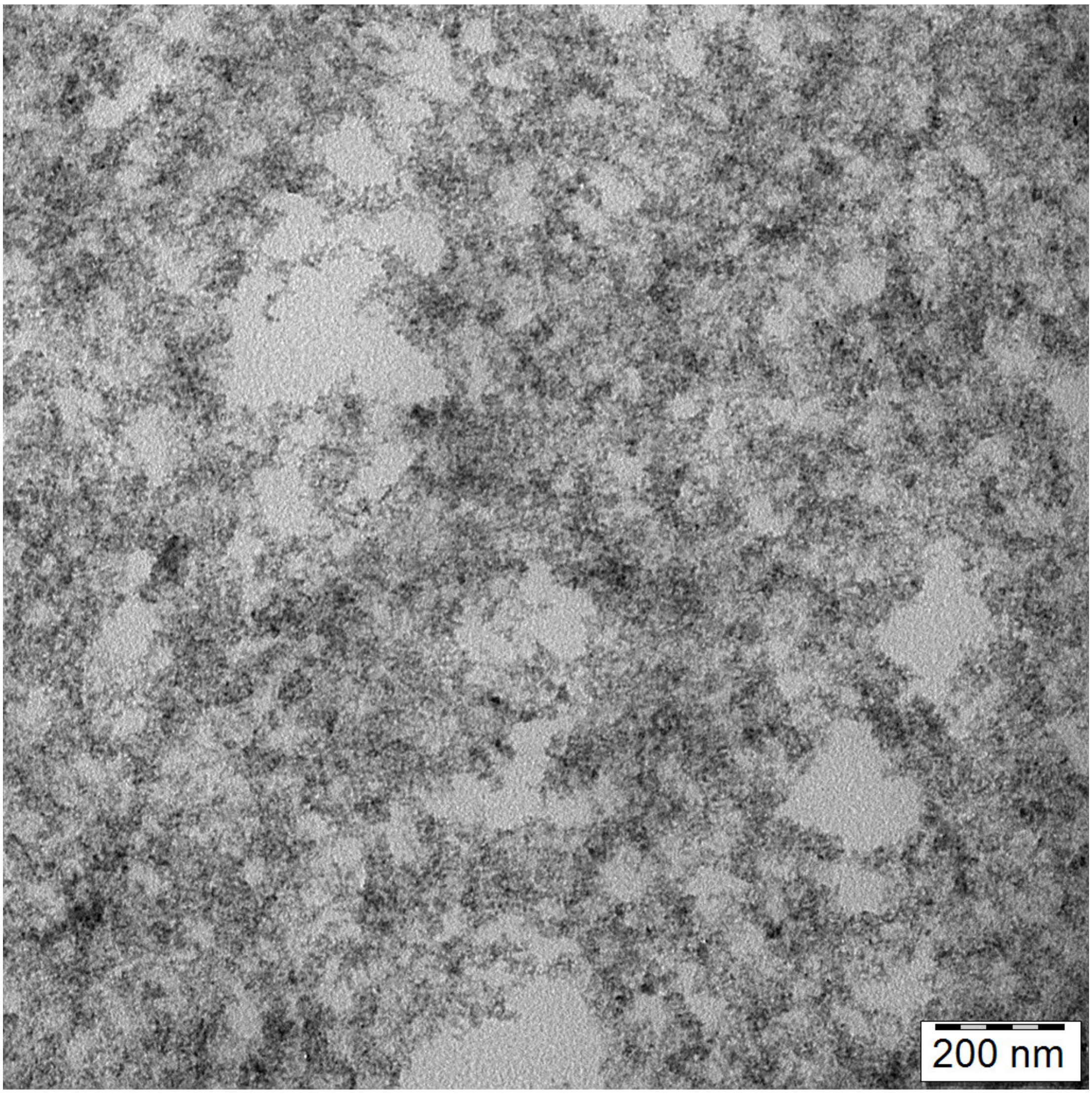


***SI-Figure S2 – multi-page (continued):*** *(c) High-resolution version of the TEM image of 0R-15T.*

***SI-Figure S2 – multi-page (continued):***

# 4R-0T

**(d)**

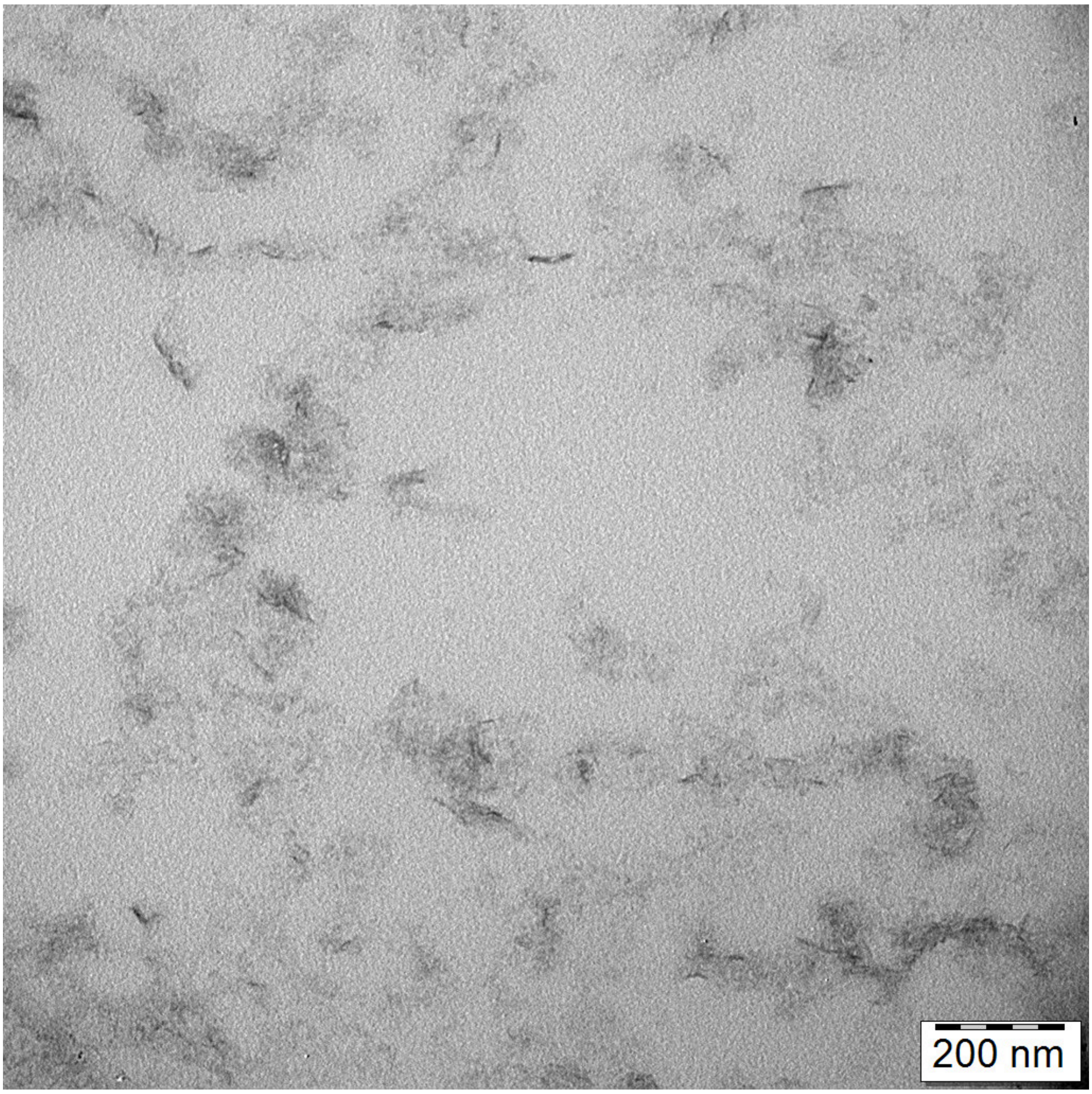


***SI-Figure S2 – multi-page (continued):*** *(d) High-resolution version of the TEM image of 4R-0T.*

***SI-Figure S2 – multi-page (continued):***

# 4R-0T, **larger-scale-view**

**(d2)**

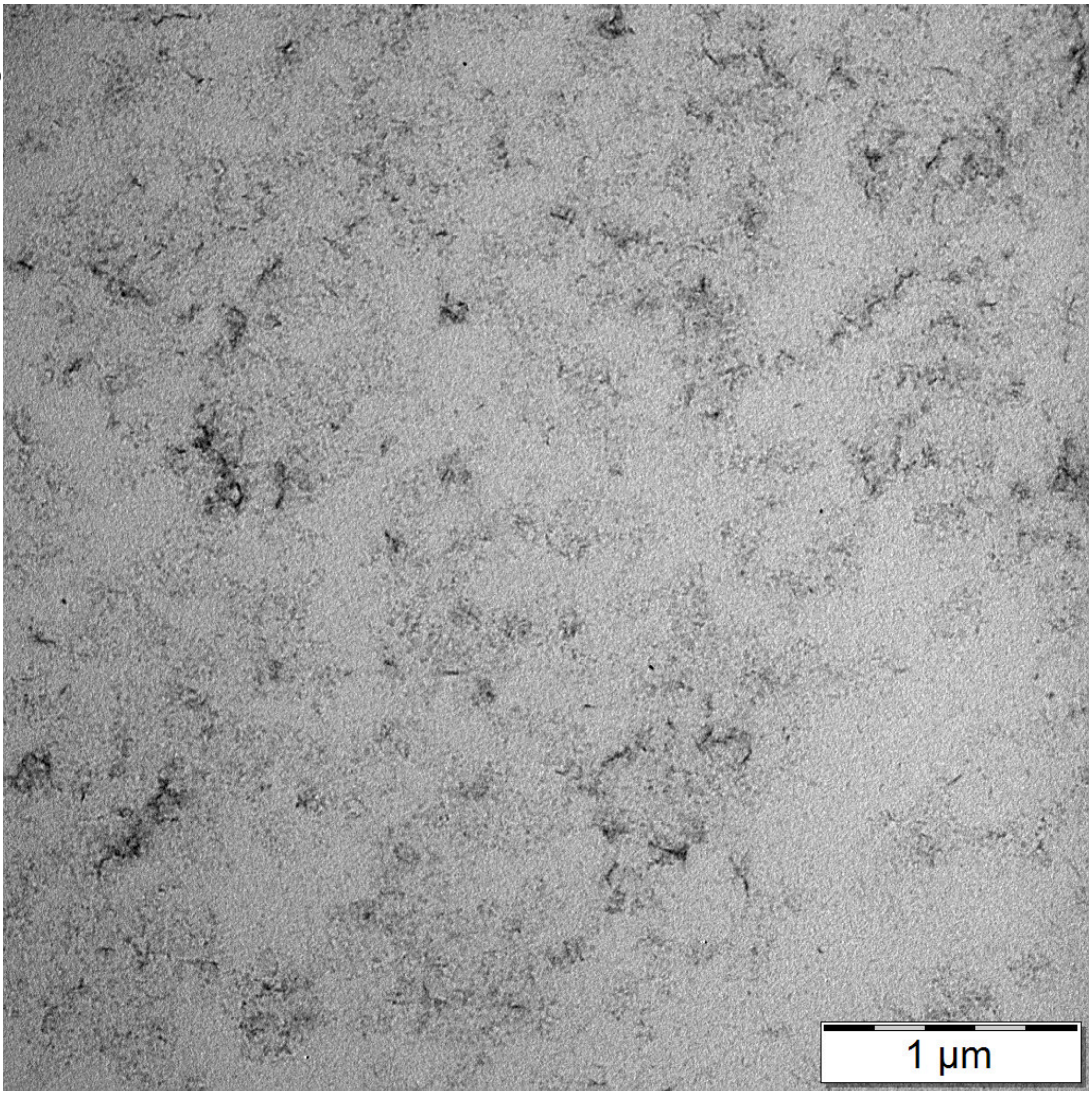


***SI-Figure S2 – multi-page (continued):*** *(d2) TEM: larger-scale-view of 4R-0T.*

***SI-Figure S2 – multi-page (continued):***

# 4R-5T

**(e)**

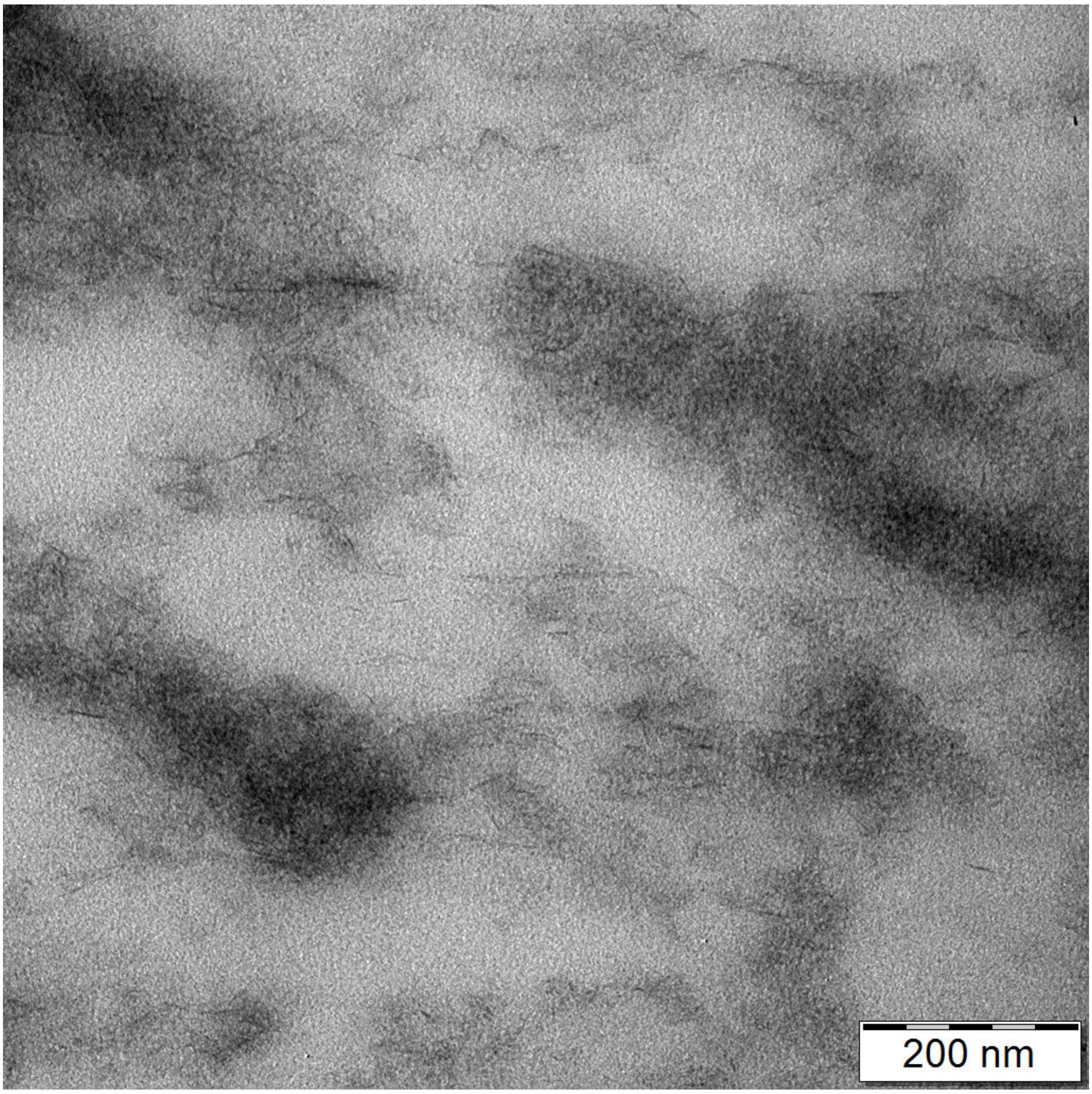


***SI-Figure S2 – multi-page (continued):*** *(e) High-resolution version of the TEM image of 4R-5T.*

***SI-Figure S2 – multi-page (continued):***

# 4R-5T, **larger-scale-view**

**(e2)**

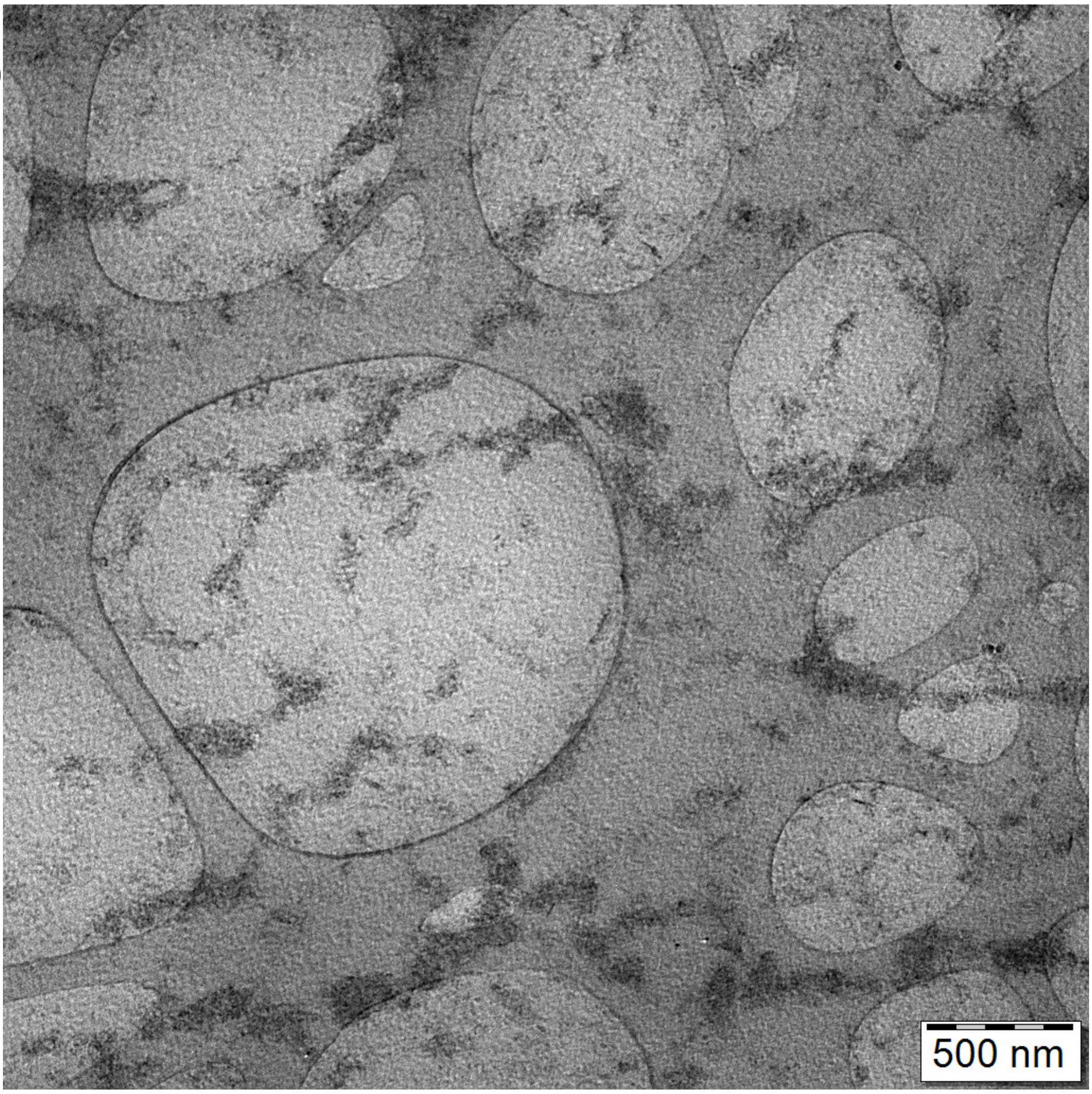


***SI-Figure S2 – multi-page (continued):*** *(e2) TEM: larger-scale-view of 4R-5T; note (!): the visible large 'pores' or 'bubbles' are not related to the sample itself: this are pores in the graphite substrate which was used to support the observed specimen.*

***SI-Figure S2 – multi-page (continued):***

# 4R-15T

**(f)**

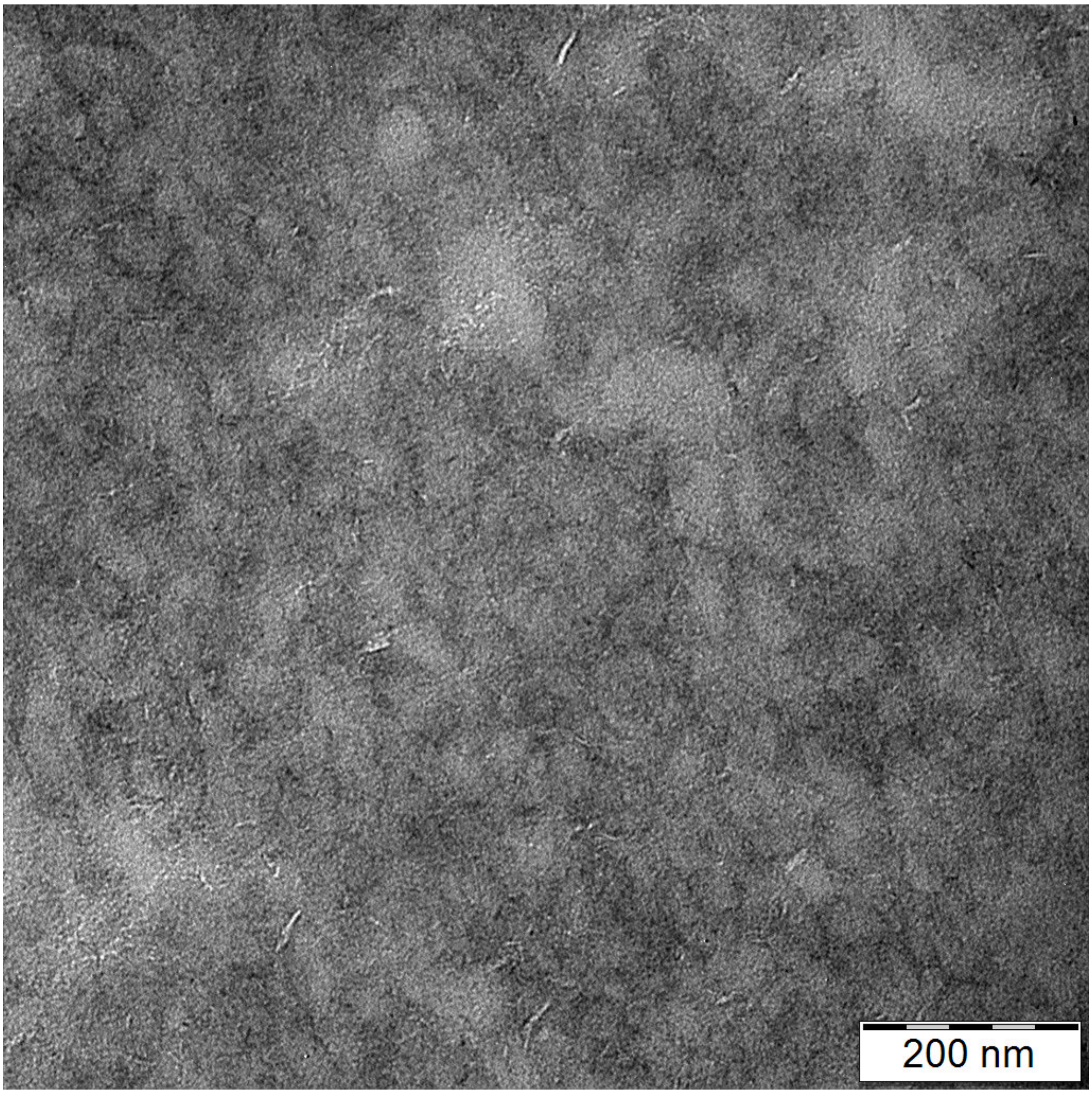


***SI-Figure S2 – multi-page (continued):*** *(f) High-resolution version of the TEM image of 4R-15T.*

***SI-Figure S2 – multi-page (continued, last image):***

# 4R-15T, **larger-scale-view**

**(f2)**

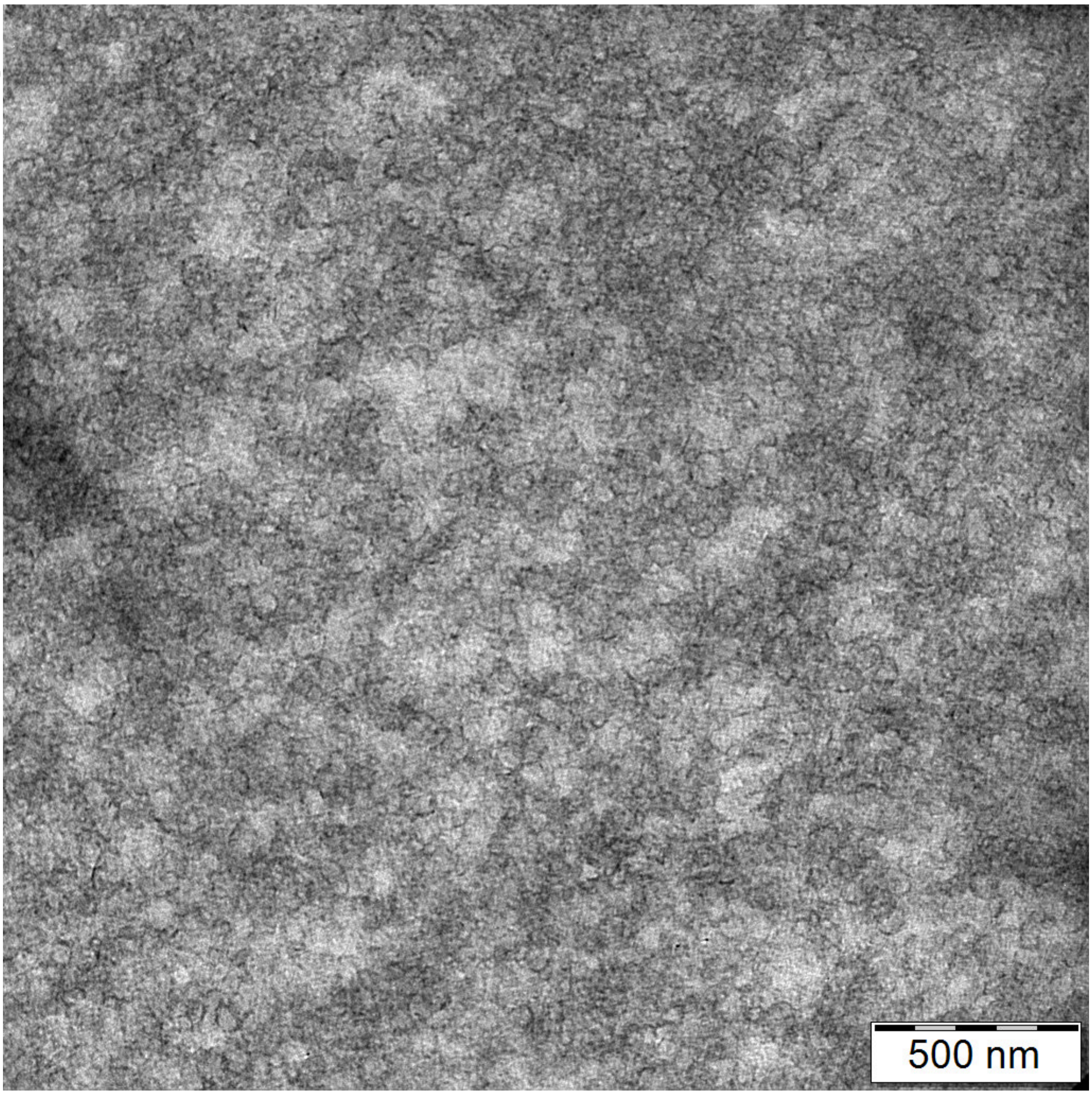


***SI-Figure S2 – multi-page (continued, last image):*** *(f2) ) TEM: larger-scale-view of 4R-15T.*

# X-ray diffraction (Morphology): detailse view of SAXS region

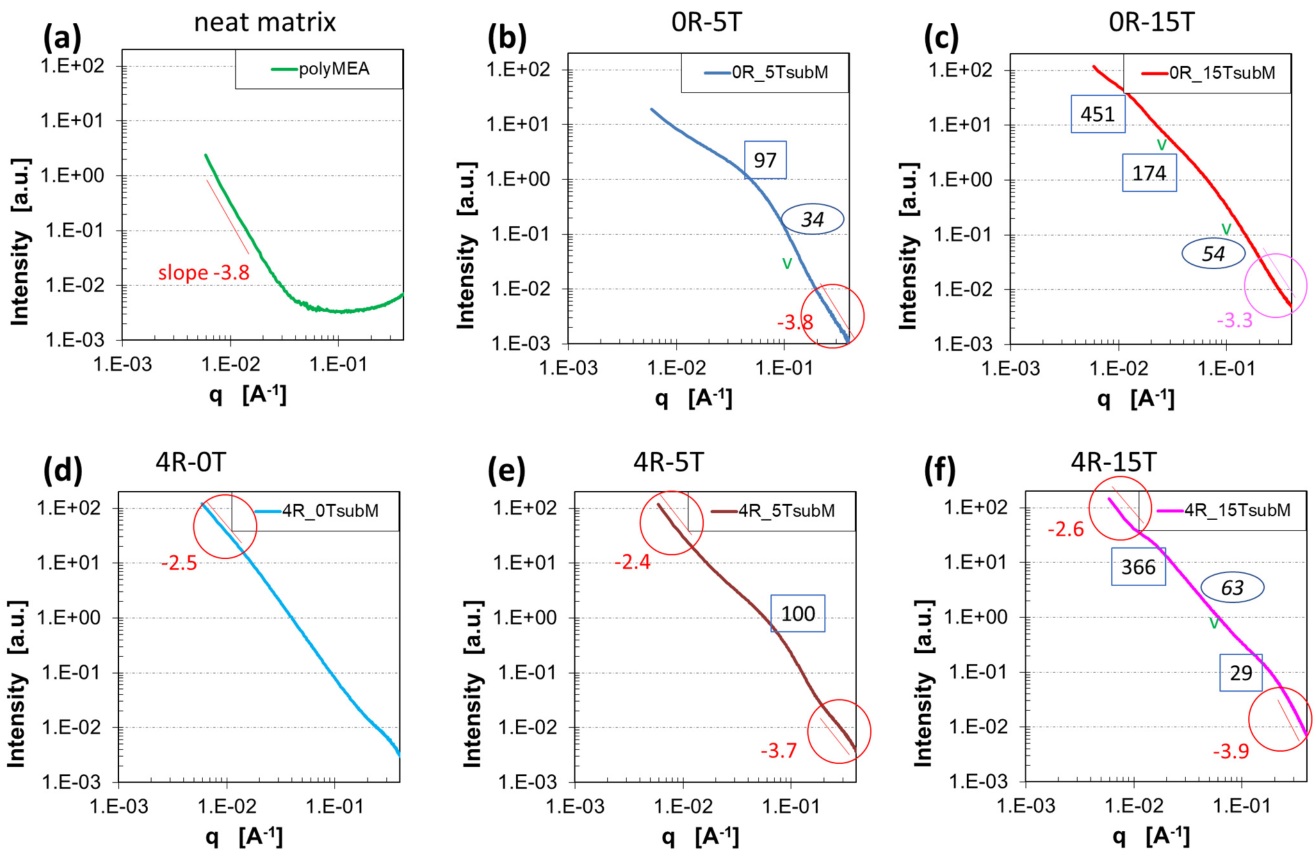


***SI-Figure S3:*** *Details of X-Ray scattering patterns after subtraction of matrix intensity: (a) neat polyMEA; (b) polyMEA + 5% of SiO2; (c) with 15% of SiO2; (d) with 4% of clay; (e) with 4% of clay + 5% of SiO2; (f) with 4% of clay + 15% of SiO2; labels: calculated characteristic distances (attempted subtraction of both polyMEA and Clay is illustrated below, in **SI-Figure S4**).*

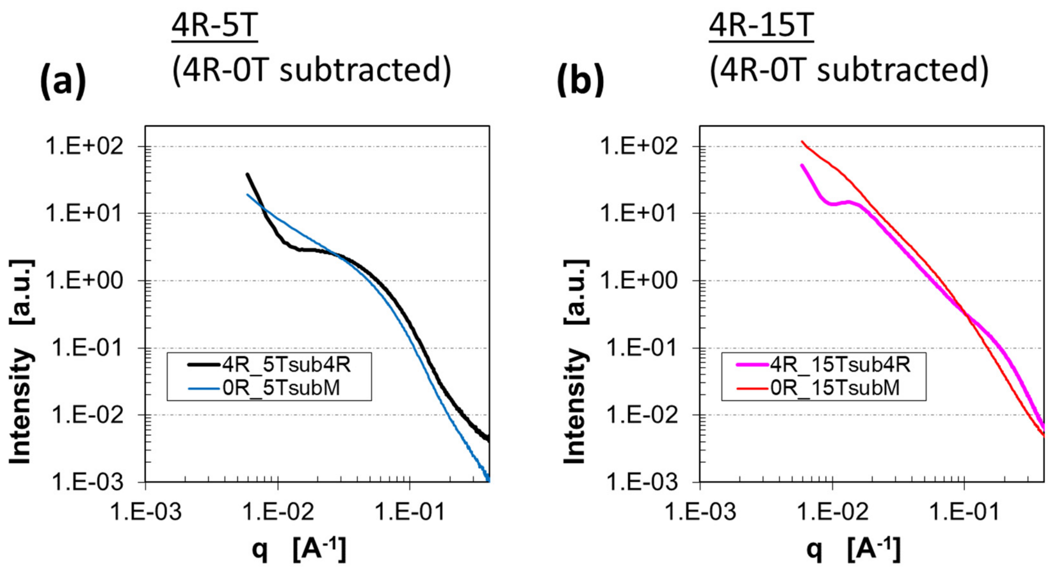


***SI-Figure S4:*** *Patterns of the SAXS region of the doubly filled samples 4R-5T and 4R-15T obtained by subtraction of the curve of the clay-containing matrix (4R-0T) from the original curves of the mentioned samples; the results suggest, that the fillers are not distributed independently of each other, which leads to strange shapes of the curves after subtraction.*

# 2. Tensile properties

## Shear moduli at small deformation after preparation and different treatment

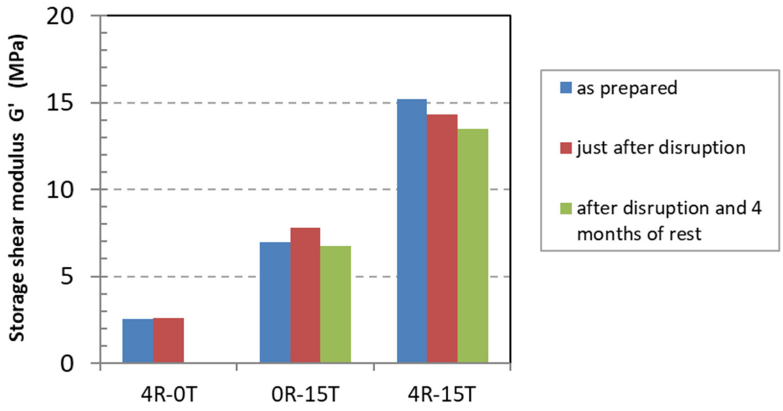


***SI-Figure S5:*** *Shear moduli at small deformation (as determined in torsion experiments), for the most important nanocomposite samples, in different states: (blue columns) as prepared; (red columns) just after disruption in tensile experiment; (green columns) after tensile disruption and subsequent 4-month-rest; corresponding Young's moduli can be calculated as E = 3 G.*

## Hysteresis tests in the as prepared state

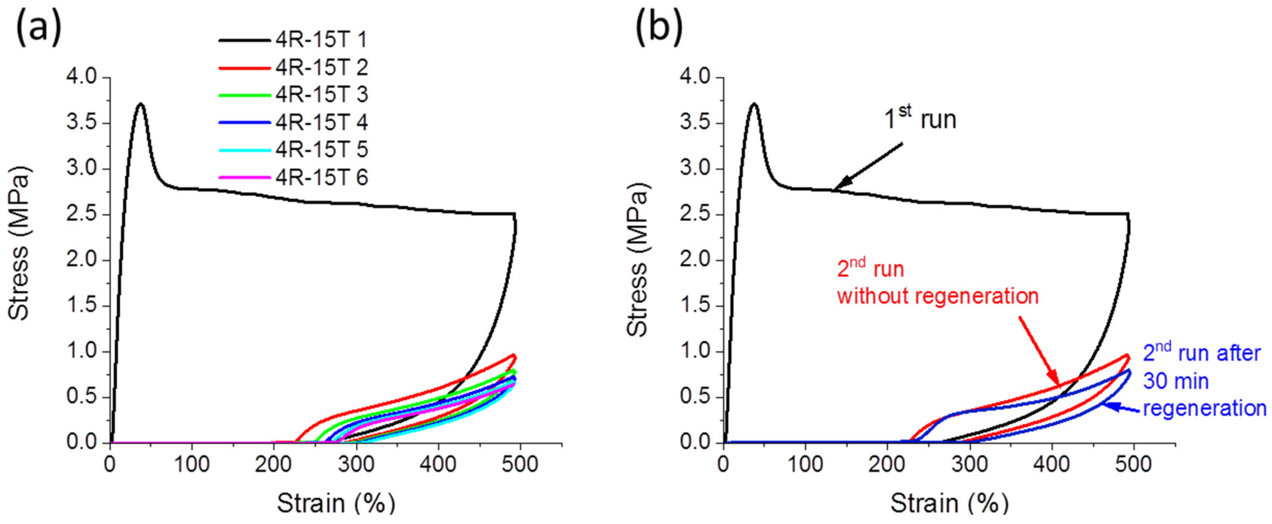


***SI-Figure S6:*** *Behaviour of the nanocomposite 4R-15T (a) upon repeated cyclic loading; (b) after a 30 min rest time following the first loading cycle.*

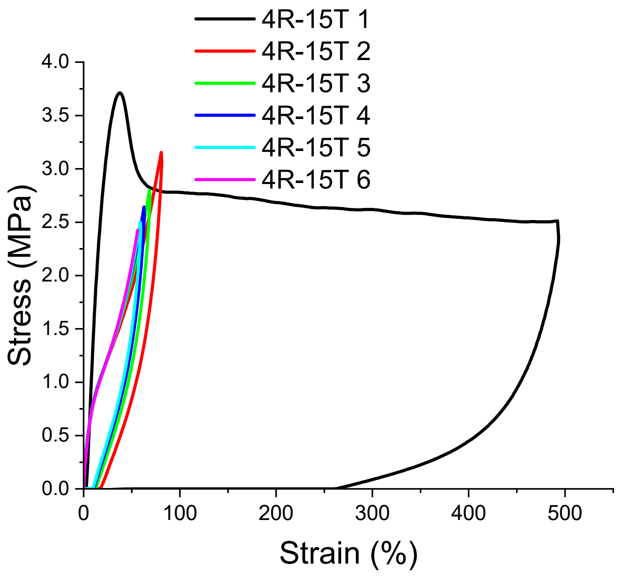


***SI-Figure S7:*** *Hysteresis behaviour of the nanocomposite 4R-15T upon repeated cyclic loading with evaluation of each cycle as a new specimen with a new initial geometry.*

# Relaxation tests

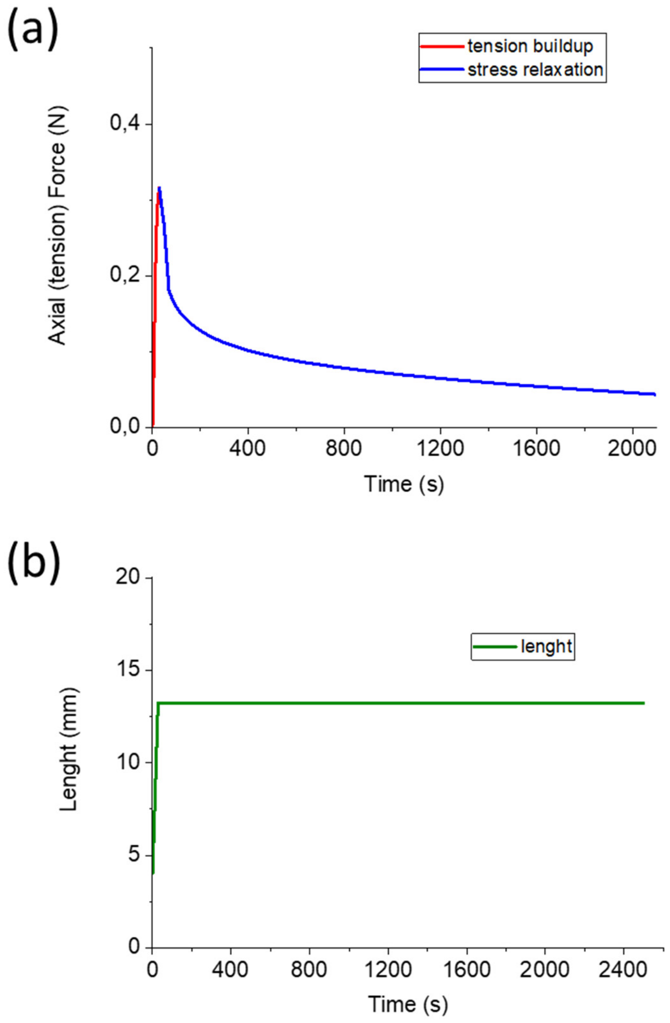


***SI-Figure S8:*** *Neat polyMEA: Stress relaxation experiment: (a) graph of stress build-up (as consequence of mechanical stretching, red line) and of subsequent relaxation (during the rest period, blue line); (b) length of the specimen between the clamps of the analysing machine during this experiment.*

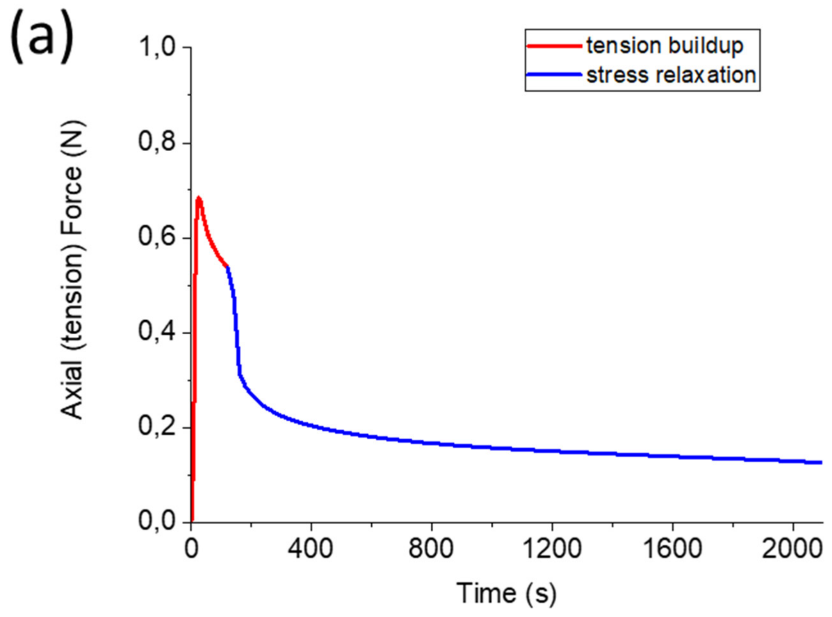


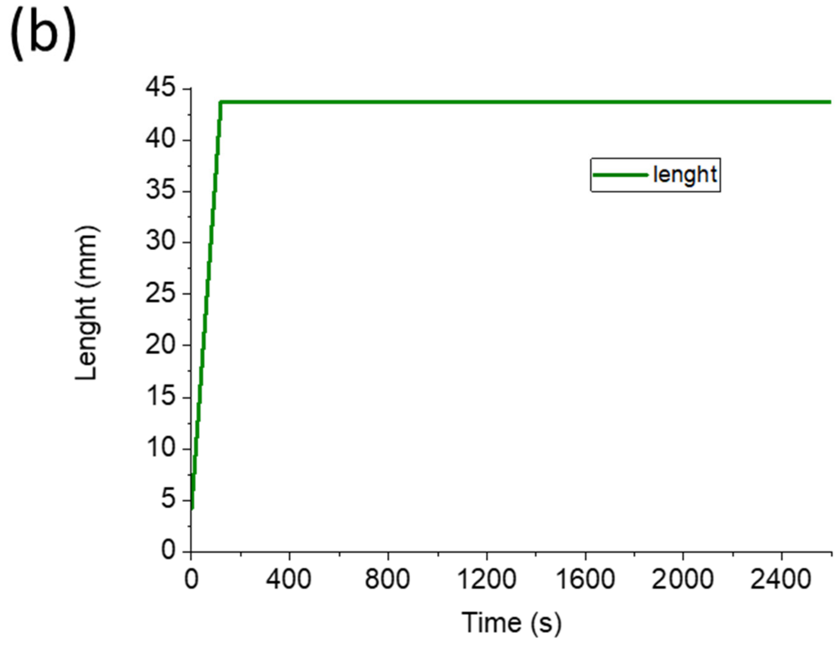


***SI-Figure S9:*** *4R-0T: Stress relaxation experiment: (a) graph of stress build-up (as consequence of mechanical stretching, red line) and of subsequent relaxation (during the rest period, blue line); (b) length of the specimen between the clamps of the analysing machine during this experiment.*

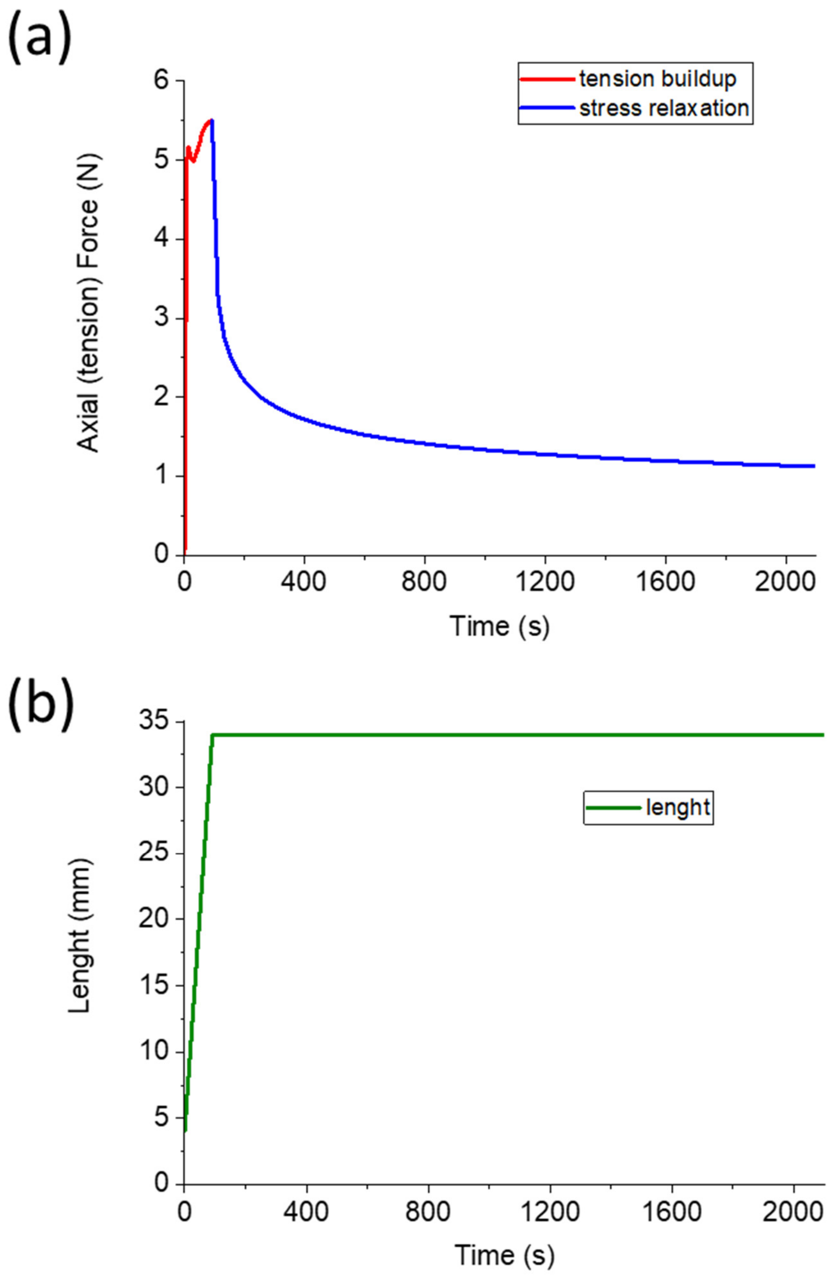


***SI-Figure S10:*** *0R-15T: Stress relaxation experiment: (a) graph of stress build-up (as consequence of mechanical stretching, red line) and of subsequent relaxation (during the rest period, blue line); (b) length of the specimen between the clamps of the analysing machine during this experiment.*

# 3. Changes after enduring very large deformations

## Tensile tests after enduring deformation until disruption

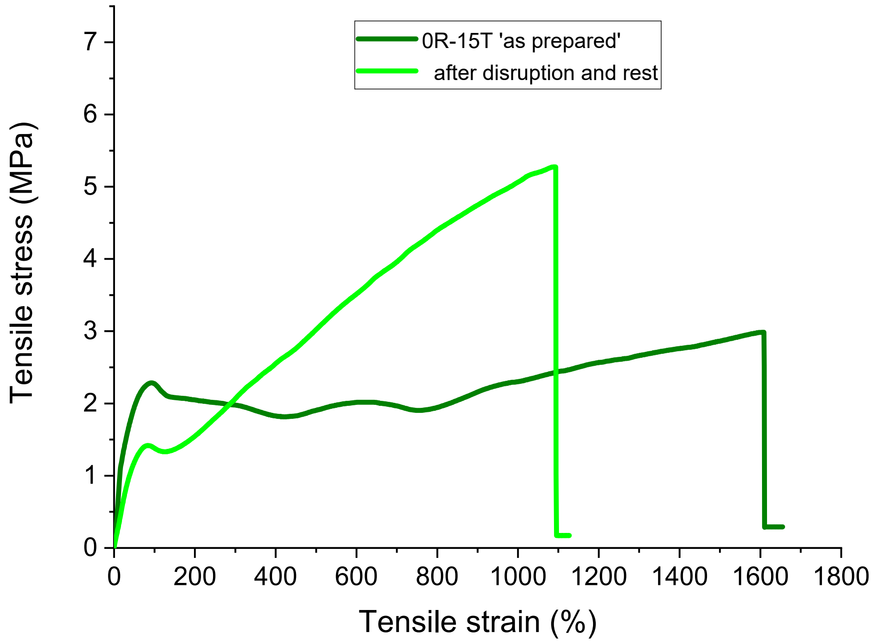


***SI-Figure S11:*** *0R-15T: successive tensile tests until disruption, first with an 'as prepared' sample, thereafter with one of the 'stretching-treated' pieces obtained by the disruption after 4 months of rest preceding the second test.*

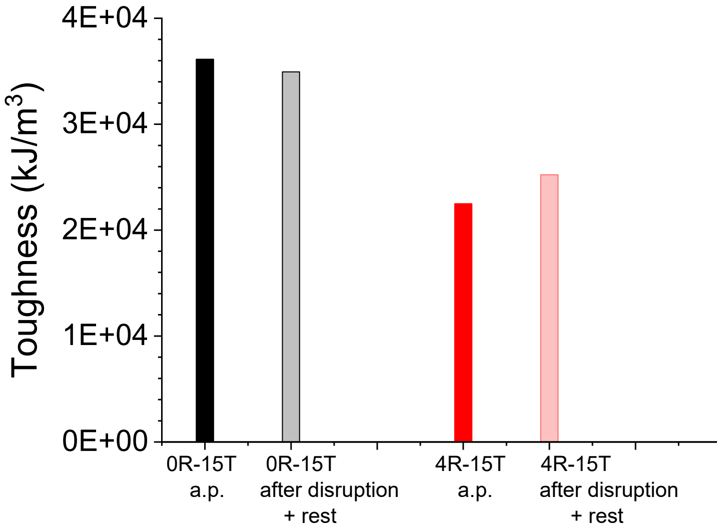


***SI-Figure S12:*** *0R-15T and 4R-15T: tensile toughness values of the nanocomposites in the 'as prepared' state (label "a.p."), and after destructive stretching and subsequent rest time of 4 months (label "after disruption + rest").*

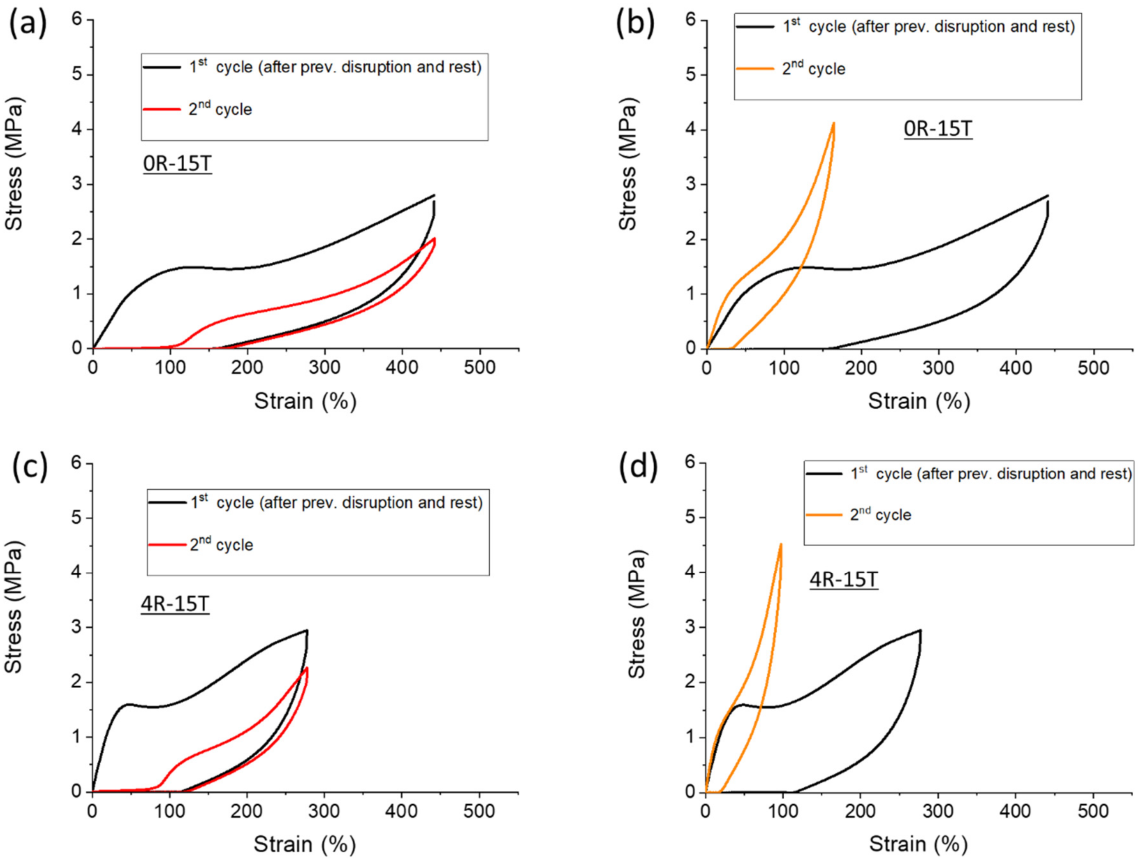


***SI-Figure S13:*** *Hysteresis curves up to large deformations of the silica-filled nanocomposites which underwent previous stretching until disruption, as well as a period of subsequent rest: (a, b) polyMEA / 15% SiO2; and (c, d) polyMEA / 4% clay / 15% SiO2: 'normal' depiction in engineering mode – second cycle evaluated using original geometry prior to first cycle (a, c); as well as evaluation of the second cycles as new specimens with new initial geometry (b, d).*

# TEM analysis of morphology changes

***SI-Figure S14 – multi-page:***

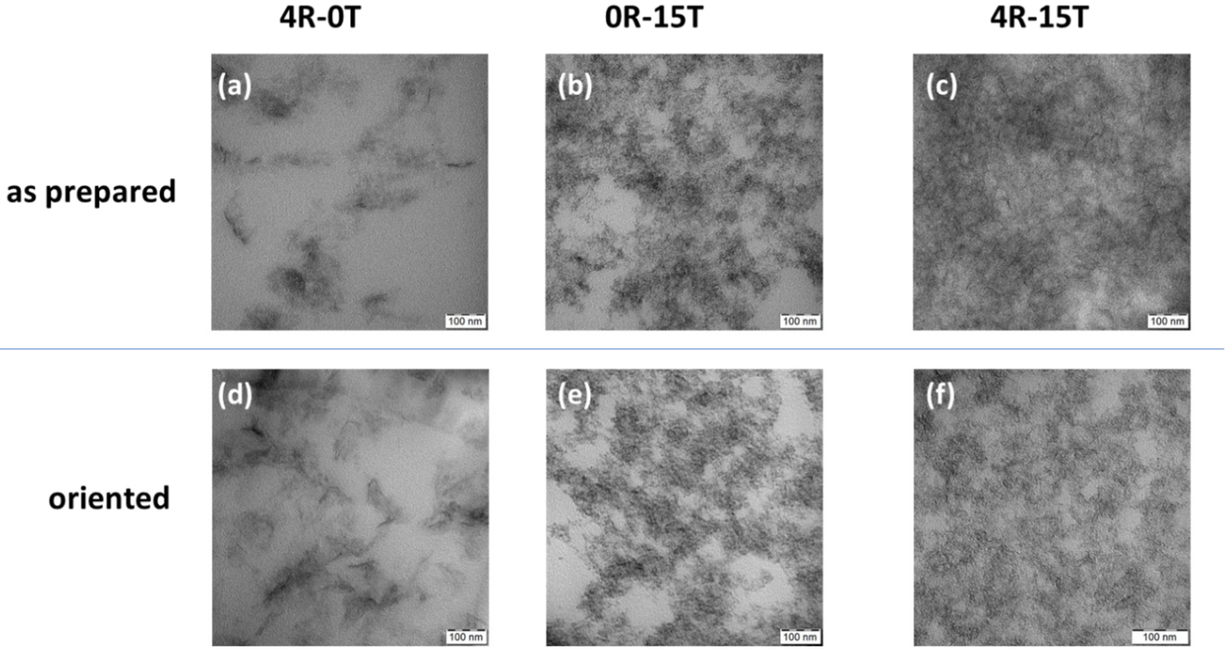


***SI-Figure S14 – multi-page:*** **Overview:** *Effect of the endured very extensive stretching (until disruption) on the morphology (TEM) of the most important nanocomposite samples (scalebar: 100 nm): (a-c) morphology in the 'as prepared' state; (d–f) morphology after a endured elongation until rupture – the elongation axis was in the image plane.*

***SI-Figure S14 – multi-page (continued):***

# 4R-0T, as prepared, intact

**(g)**

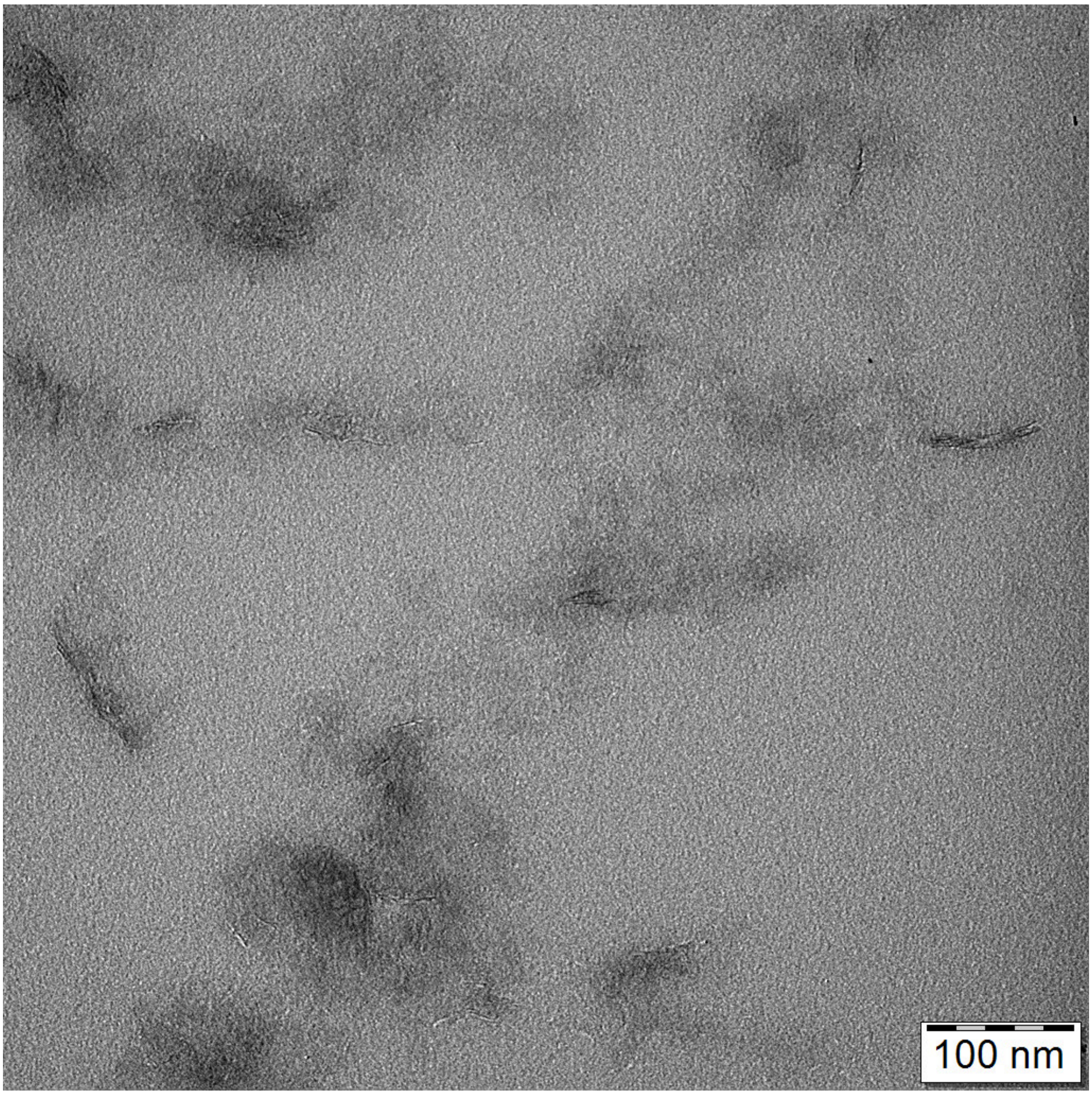


***SI-Figure S14 – multi-page (continued):*** *(g) High-resolution TEM image of* 4R-0T*, 'as prepared', intact.*

***SI-Figure S14 – multi-page (continued):***

# 0R-15T, as prepared, intact

**(h)**

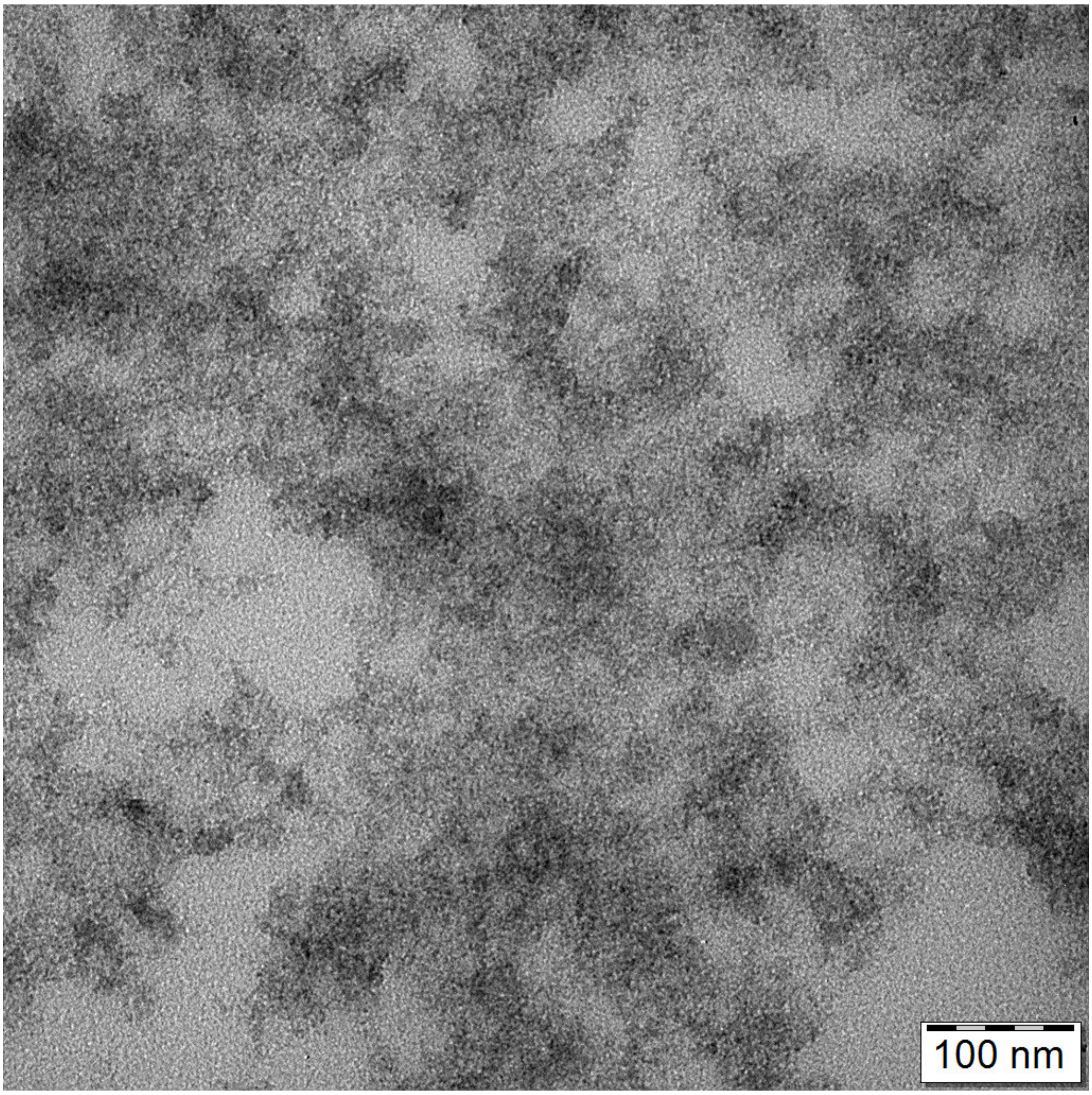


***SI-Figure S14 – multi-page (continued):*** *(h) High-resolution TEM image of* 0R-15T*, 'as prepared', intact.*

***SI-Figure S14 – multi-page (continued):***

# 4R-15T, as prepared, intact

**(i)**

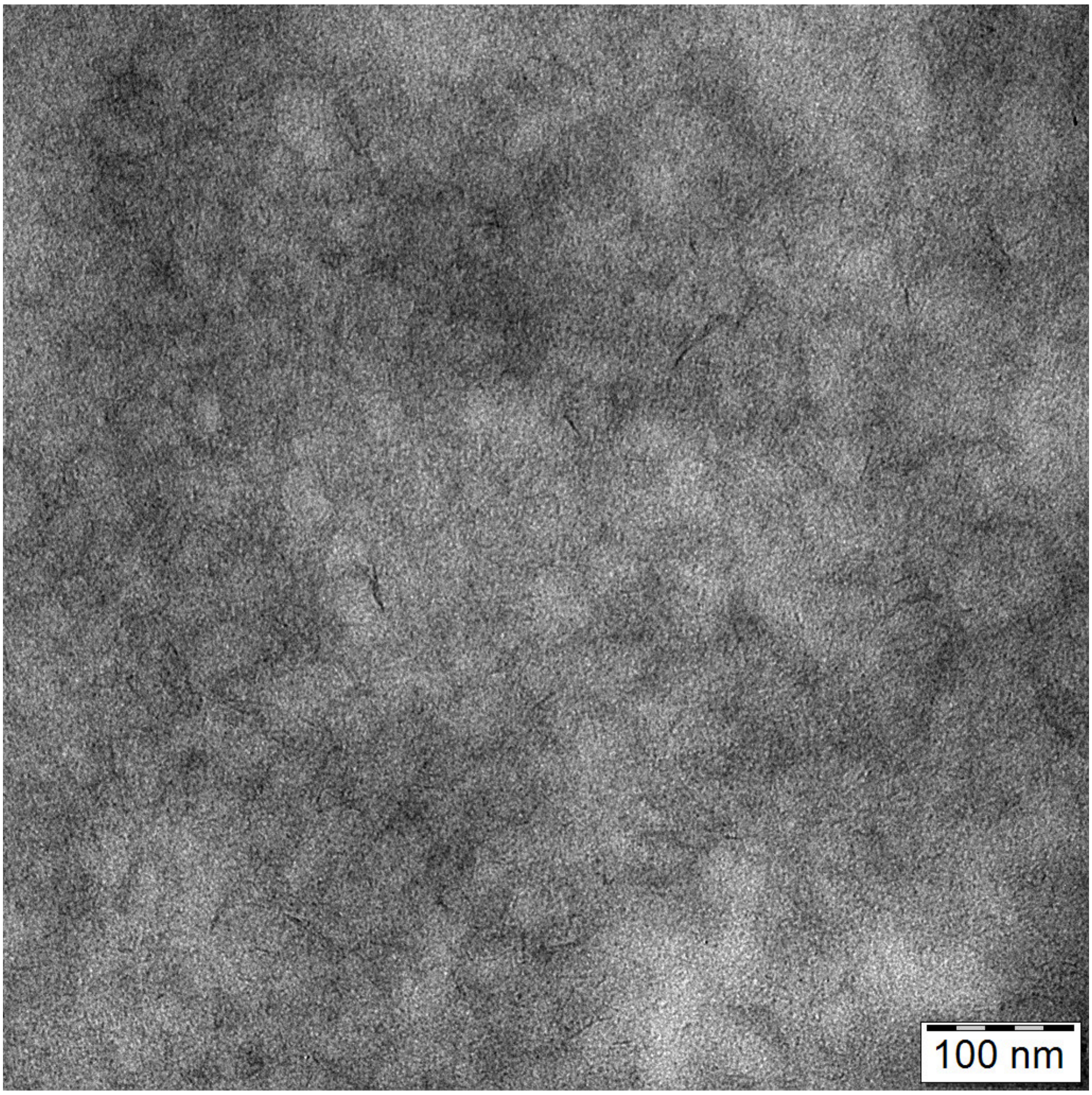


***SI-Figure S14 – multi-page (continued):*** *(i) High-resolution TEM image of* 4R-15T*, as prepared, intact.*

***SI-Figure S14 – multi-page (continued):***

# 4R-0T, after stretching until disruption

**(j)**

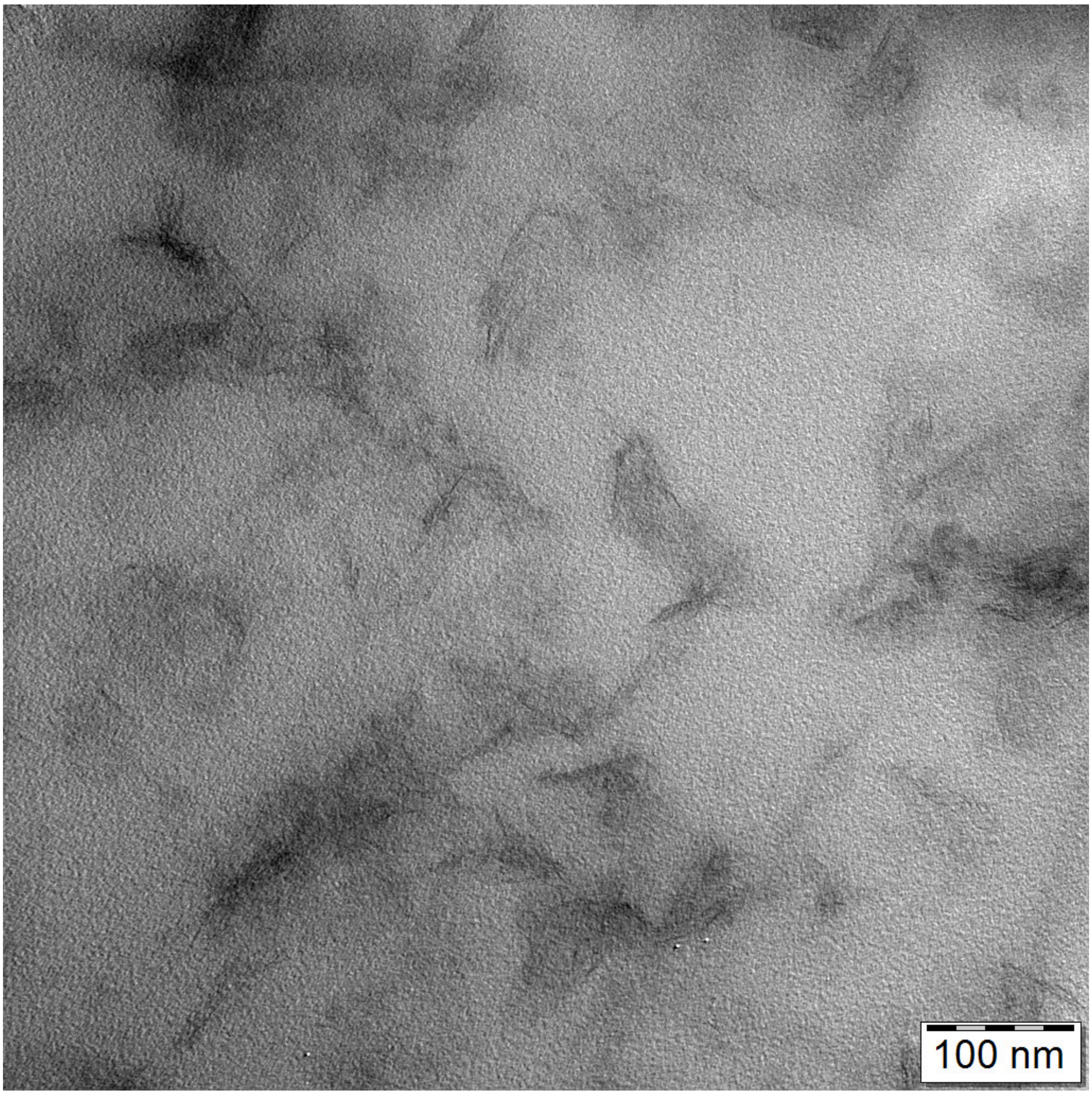


***SI-Figure S14 – multi-page (continued):*** *(j) High-resolution TEM image of* 4R-0T *after stretching until disruption – the elongation axis was in the image plane.*

***SI-Figure S14 – multi-page (continued):***

# 0R-15T, after stretching until disruption

**(k)**

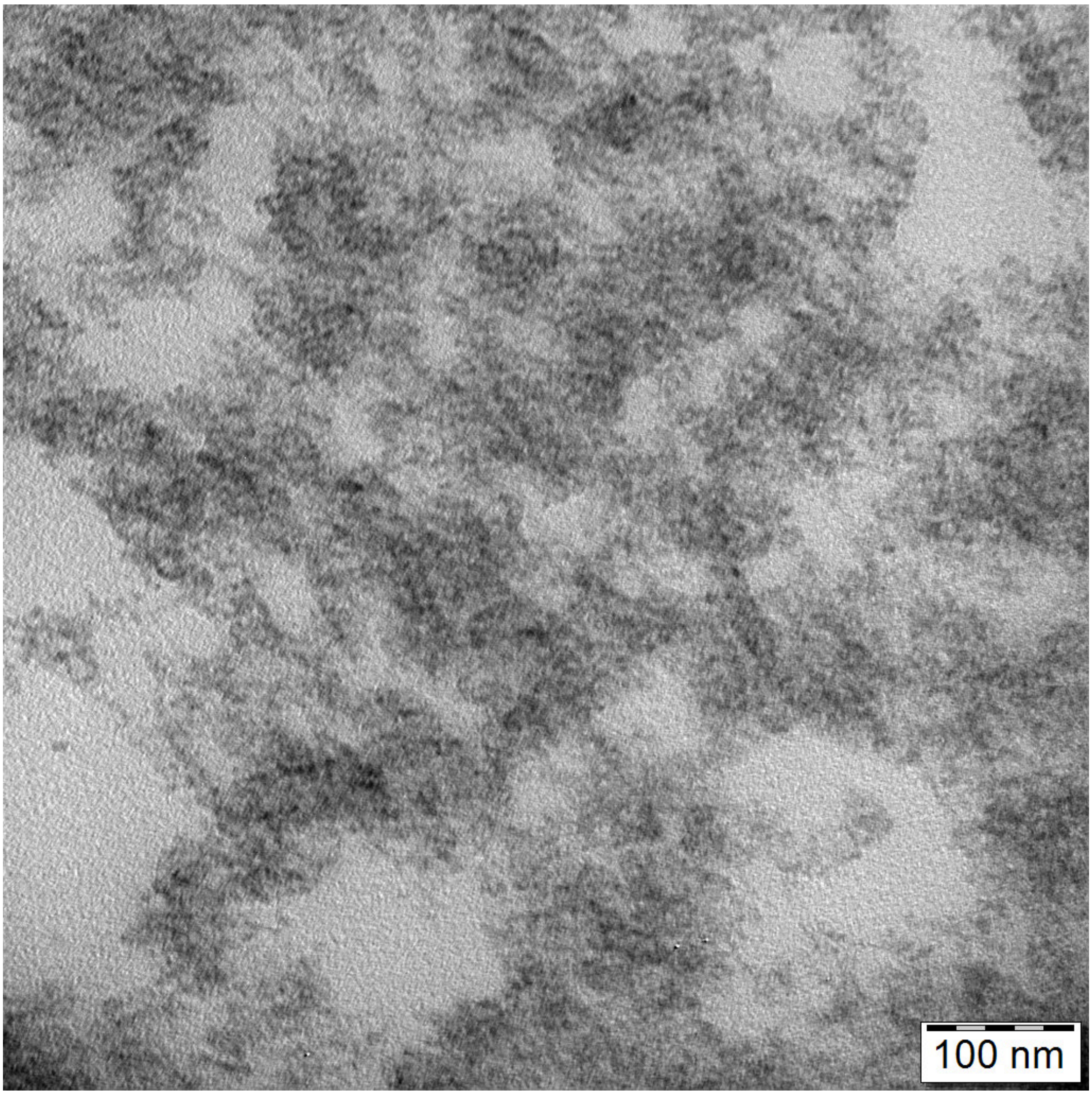


***SI-Figure S14 – multi-page (continued):*** *(k) High-resolution TEM image of* 0R-15T *after stretching until disruption – the elongation axis was in the image plane.*

***SI-Figure S14 – multi-page (continued, last image):***

# 4R-15T, after stretching until disruption

**(l)**

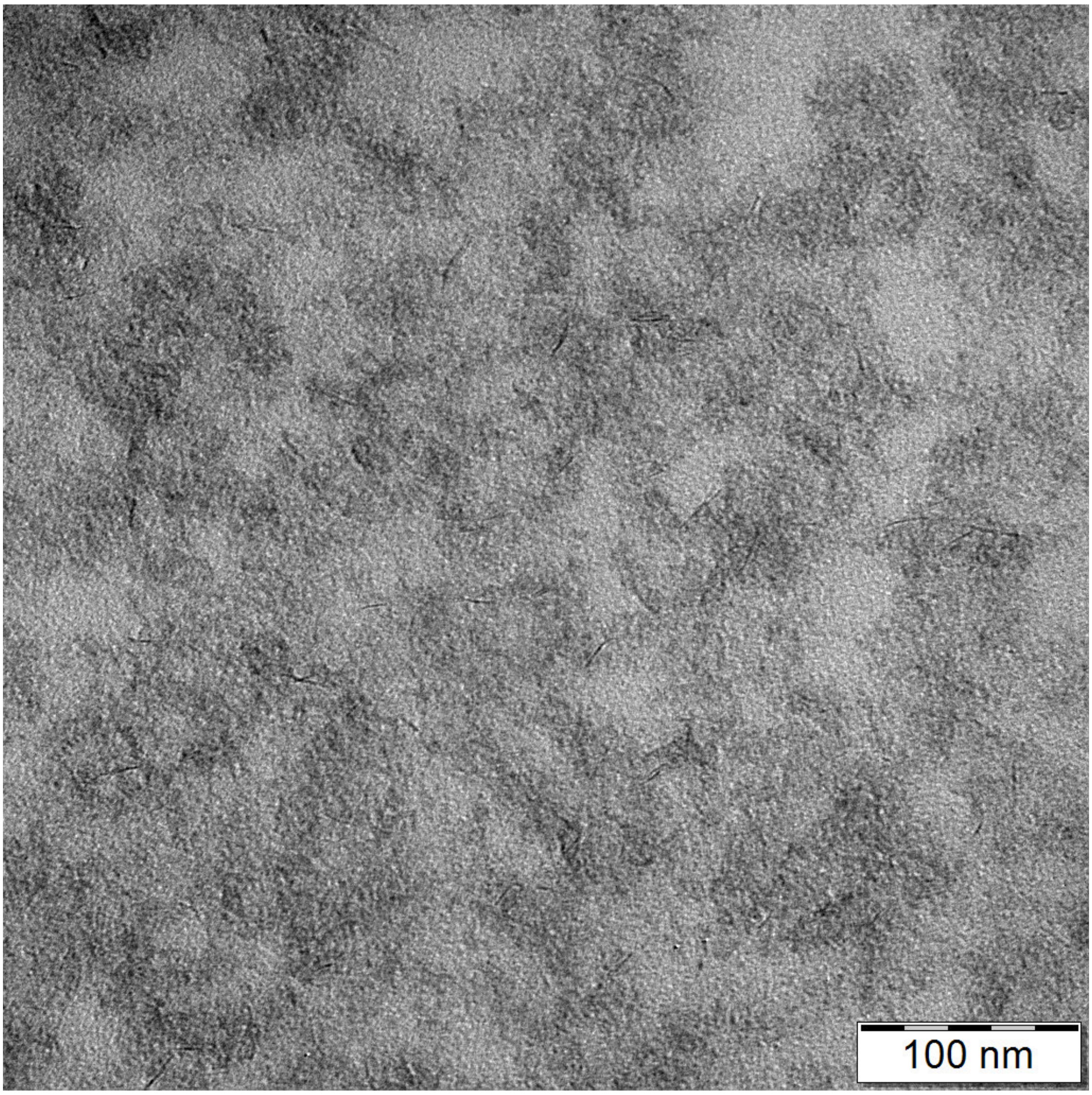


***SI-Figure S14 – multi-page (continued, last image):*** *(l) High-resolution TEM image of* 4R-15T after stretching until disruption – the elongation axis was in the image plane.

## X-ray scattering analysis of morphology changes

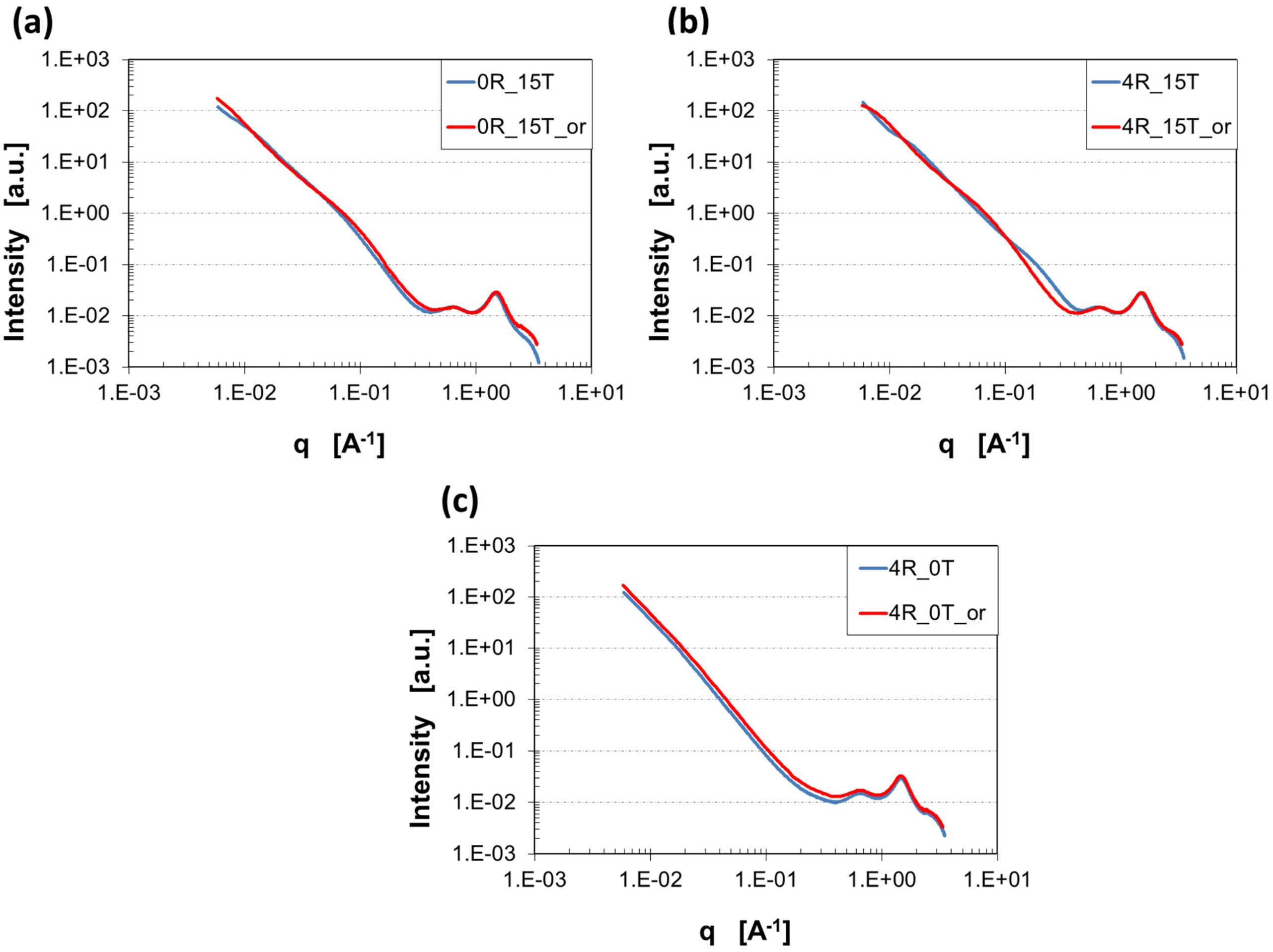


***SI-Figure S15:*** *Effect of previous elongation until destruction on some of the X-ray diffraction patterns of the most important among the studied elastomers: (a) sample with 15 wt.% of nano-SiO2*

## 2-dimensional X-ray diffractograms before and after disruption test

***SI-Figure S16 – multi-page:***

(a)

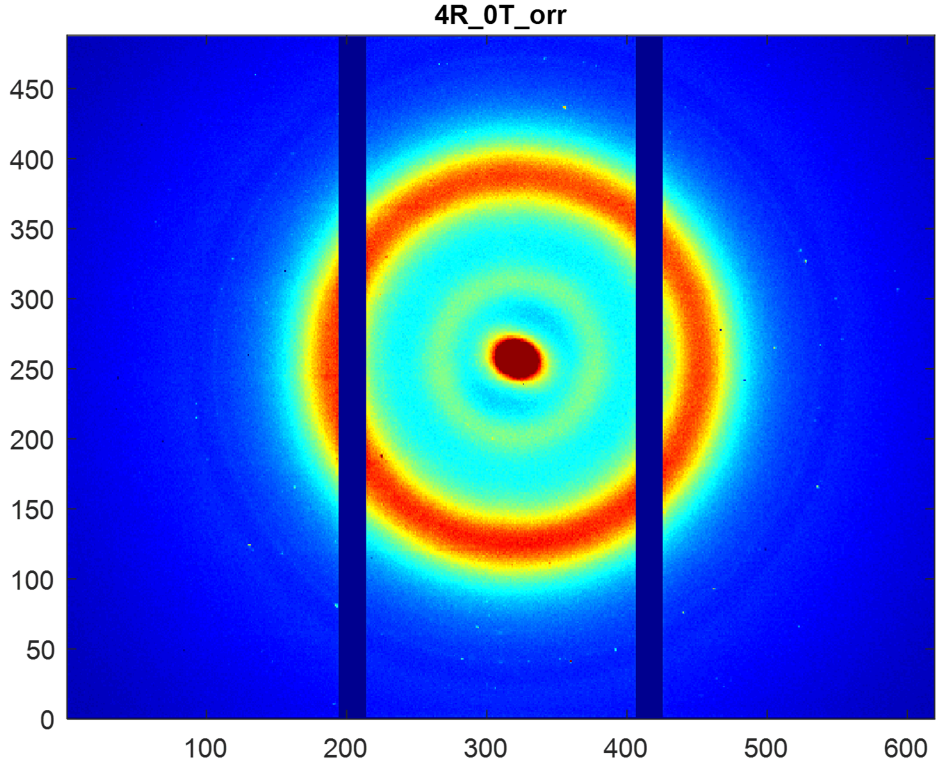


(b)

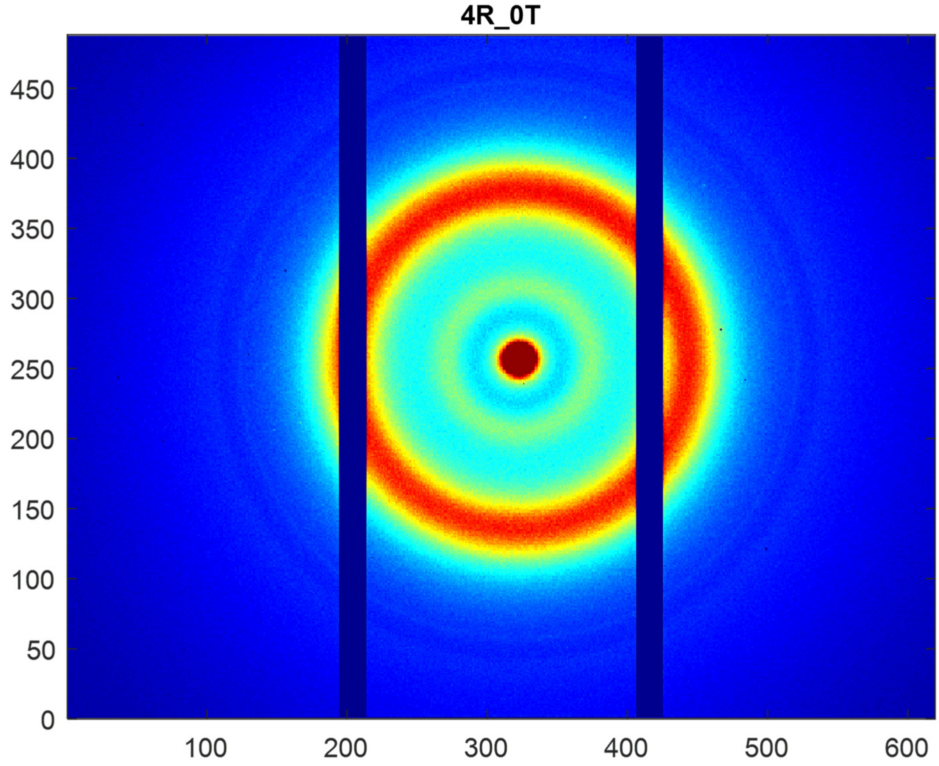


***SI-Figure S16 – multi-page:*** *2D diffractograms of the nanocomposite 4R-0T (4 wt.% of clay as the exclusive filler): (a) in <u>partly aligned</u> state ca. 30 min after tensile test until disruption; (b) in the intact 'as prepared' state.*

***SI-Figure S16 – multi-page (continued):***

(c)

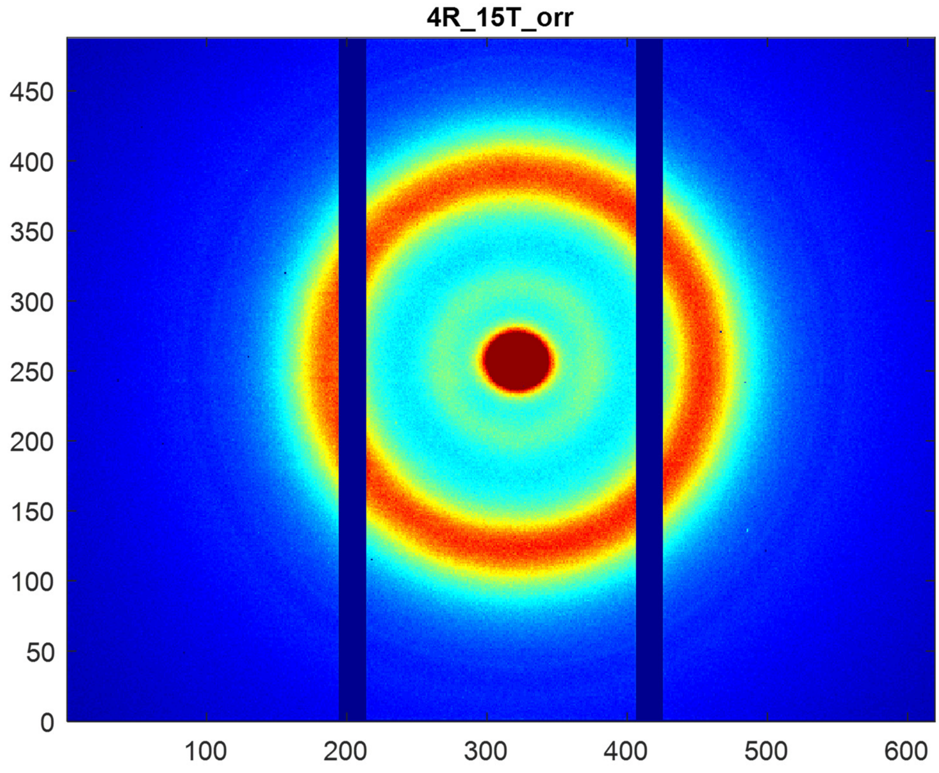


(d)

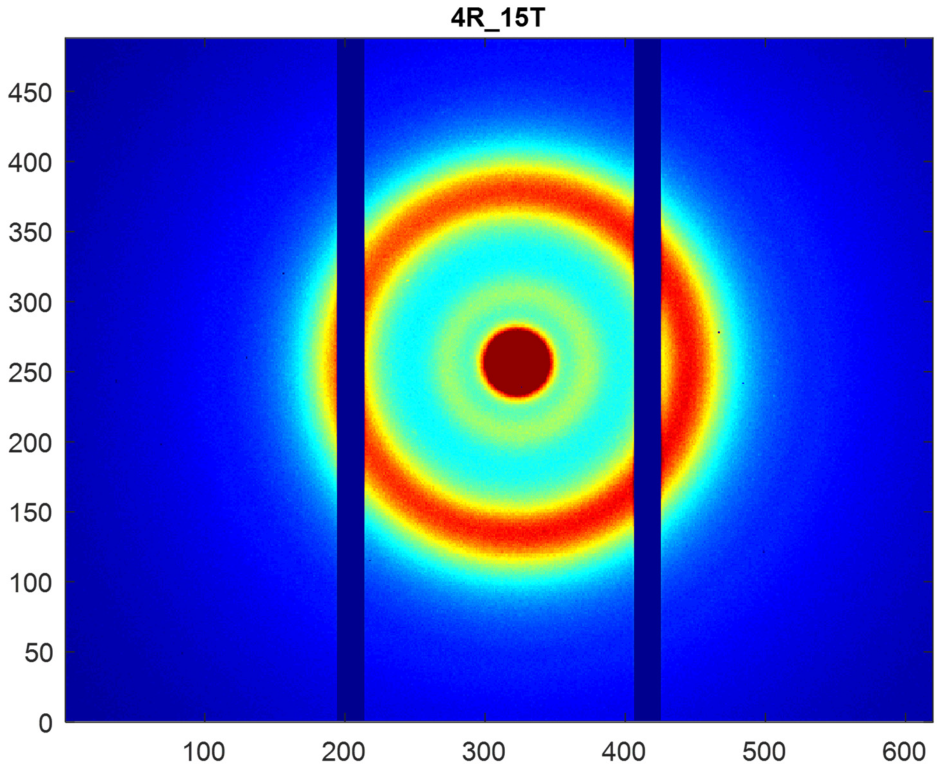


***SI-Figure S16 – multi-page (continued):*** *2D diffractograms of the nanocomposite 4R-15T (4 wt.% of clay + 15 wt.% of silica): (c) in* *__slightly anisotropic__* *state ca. 30 min after tensile test until disruption; (d) in the intact 'as prepared' state.*

***SI-Figure S16 – multi-page (continued):***

(e)

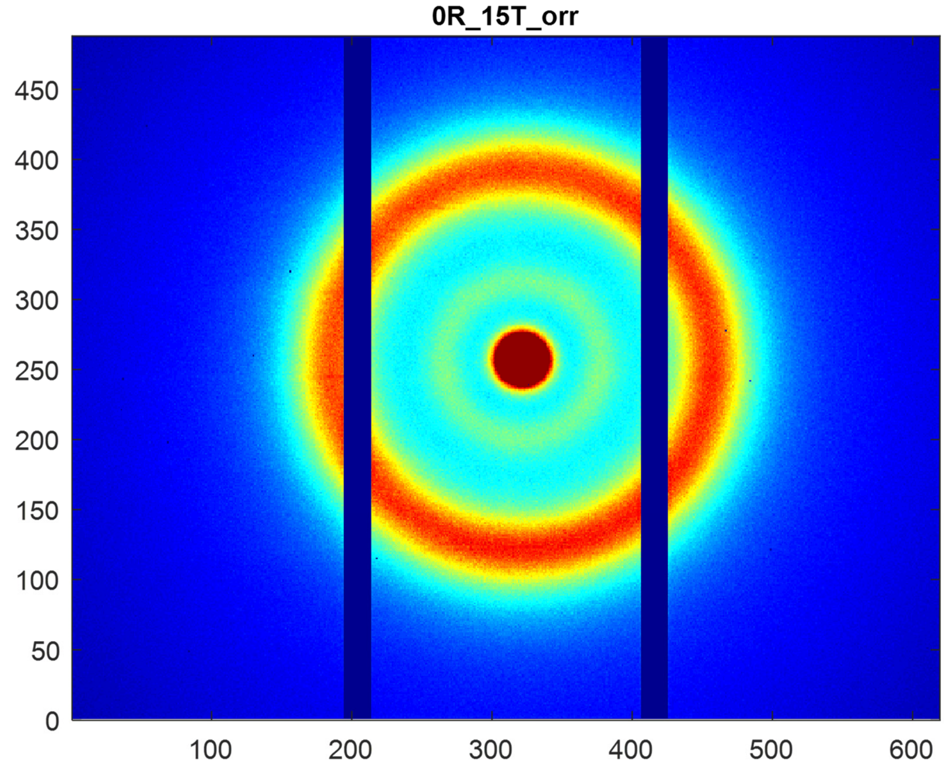


(f)

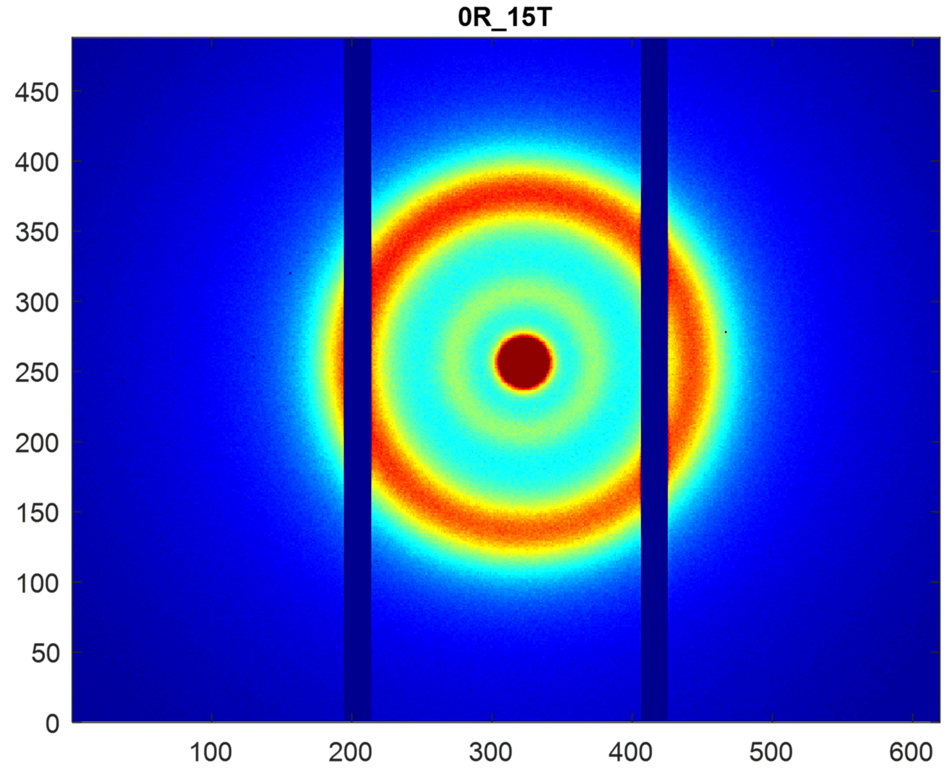


***SI-Figure S16 – multi-page (continued):*** *2D diffractograms of the nanocomposite 0R-15T (15 wt.% of silica as exclusive filler): (e) in* <u>*isotropic, non-oriented*</u> *state ca. 30 min after tensile test until disruption; (f) in the intact 'as prepared' state.*

***SI-Figure S16 – multi-page (continued, last image):***

(g)

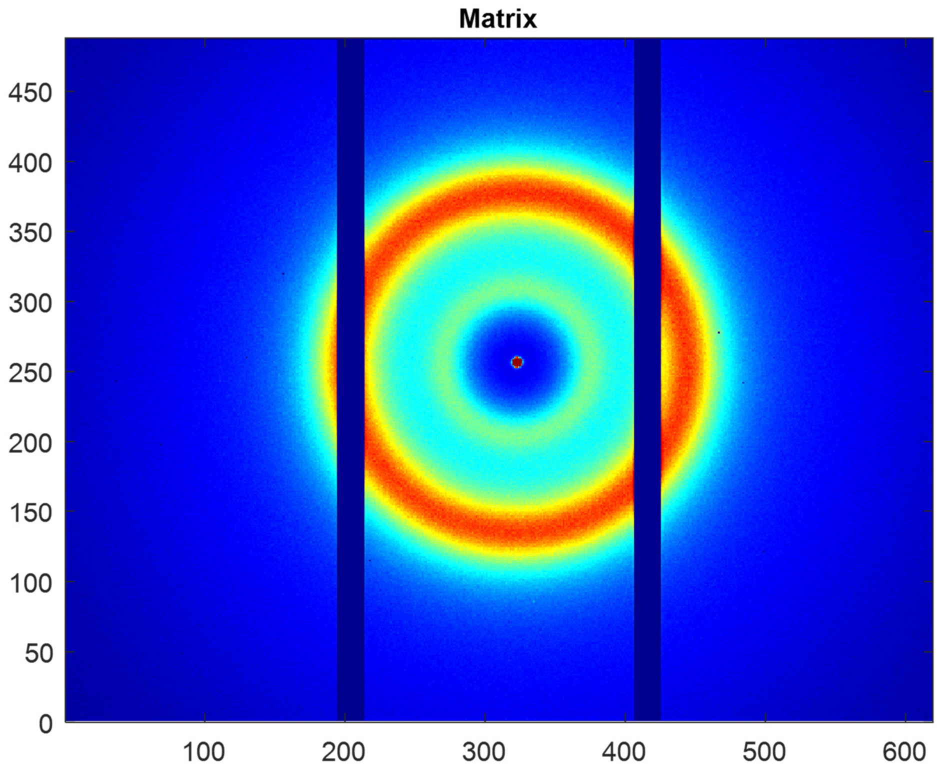


***SI-Figure S16 – multi-page (continued, last image):*** *(g) 2D diffractogram of neat polyMEA in the 'as prepared' state (as reference).*

# 4. Thermal properties (DSC)

***SI-Figure S17 – multi-page:***

**0R-15T**

(a)

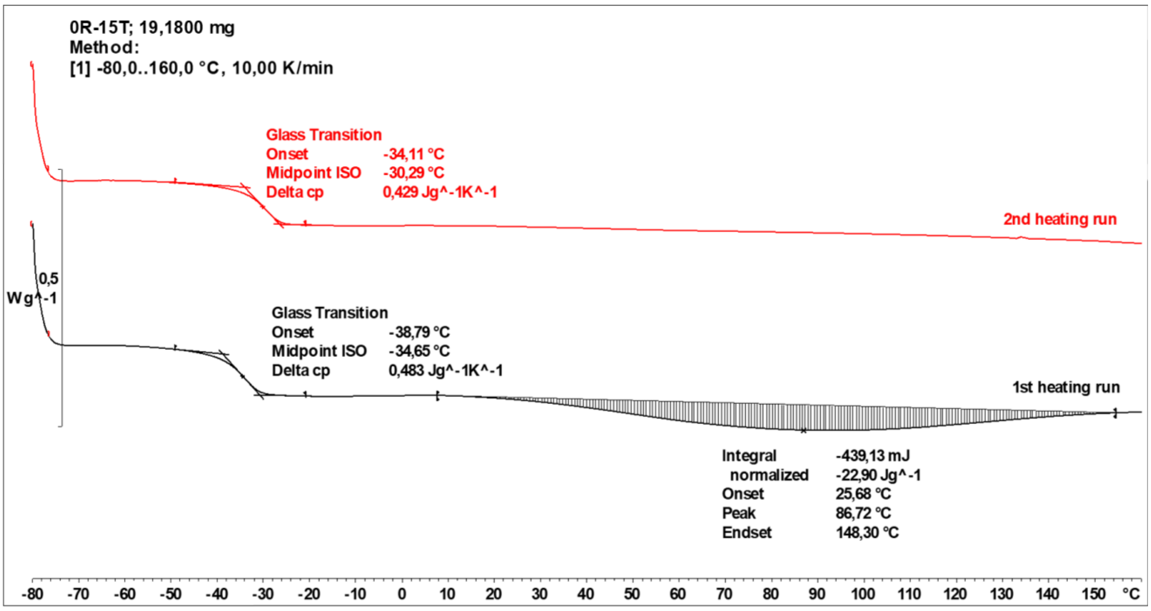


**neat polyMEA**

(b)

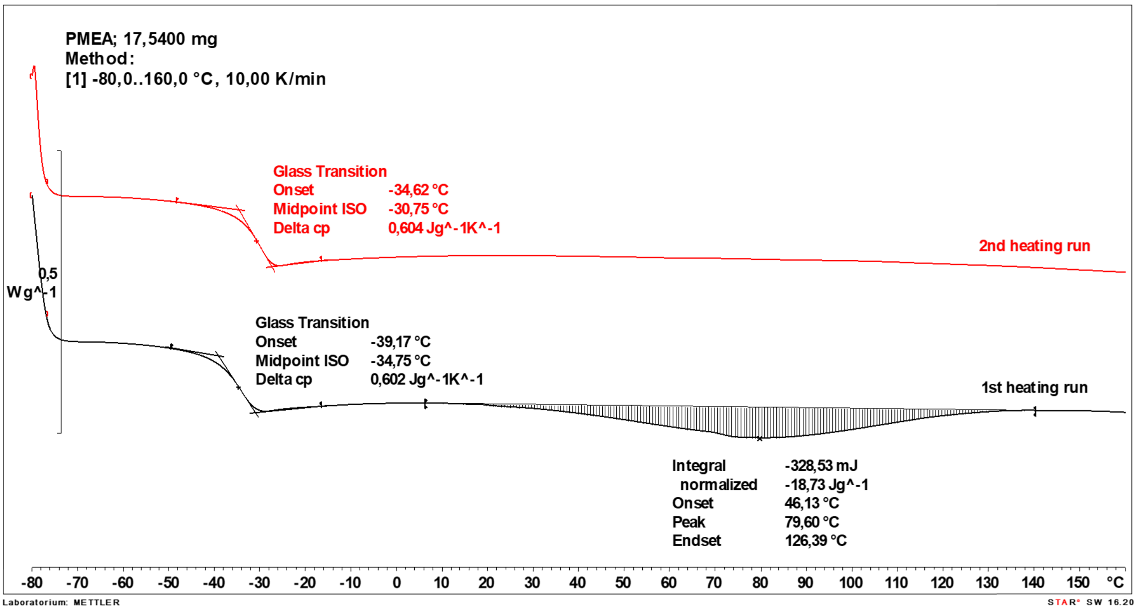


***SI-Figure S17 – multi-page:*** *DSC trace of (a) 0R-15T (polyMEA with 15 wt.% of nano-SiO2); (b) neat polyMEA matrix.*

***SI-Figure S17 – multi-page (continued):***

**4R-0T**

(c)

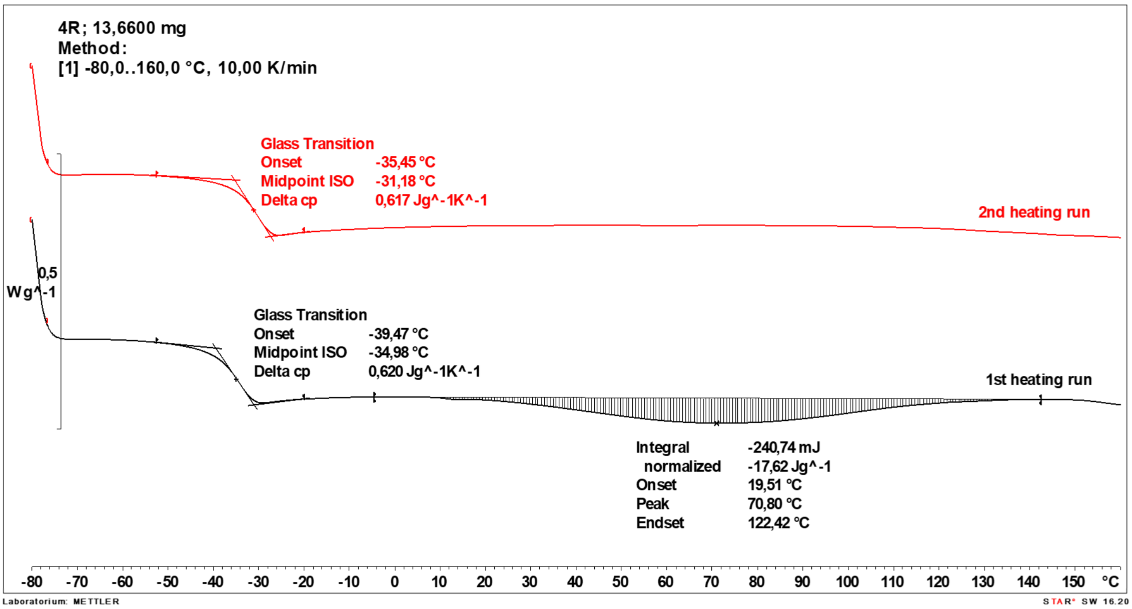


**10R-0T**

(d)

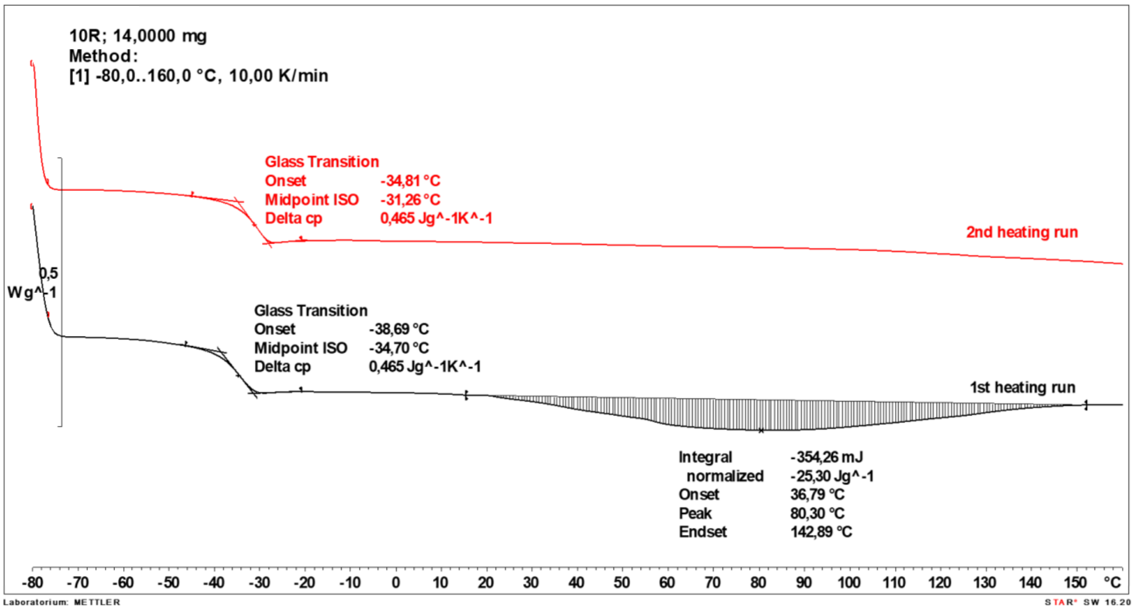


***SI-Figure S17 – multi-page:*** *DSC trace of (c) 4R-0T (polyMEA with 4 wt.% of clay); (d) 10R-0T (polyMEA with 10 wt.% of clay).*

***SI-Figure S17 – multi-page (continued):***

**0R-5T**

(e)

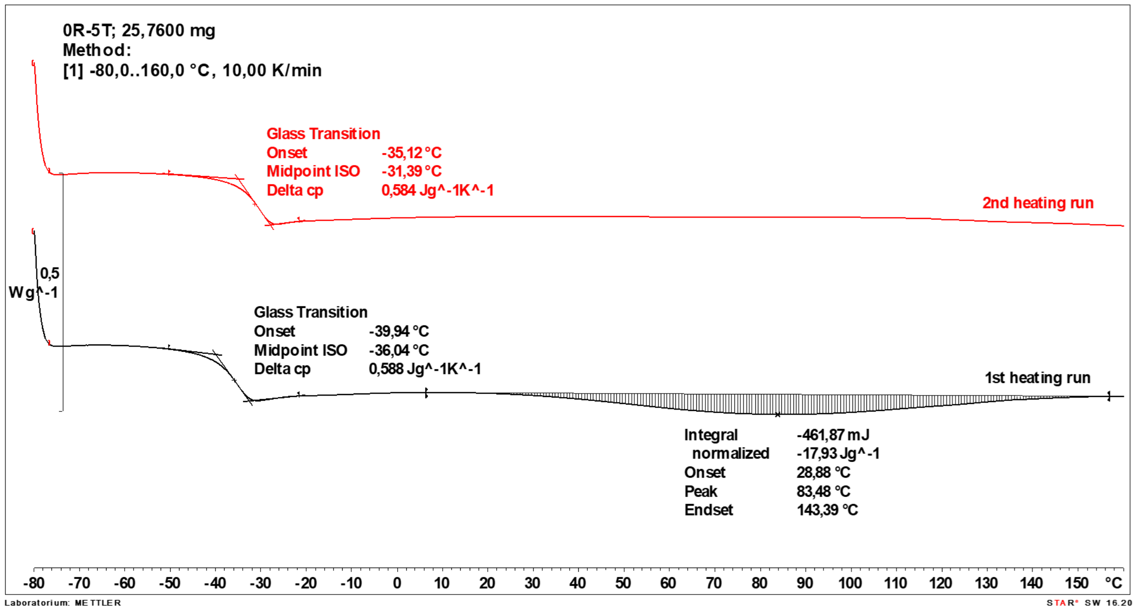


**4R-5T**

(f)

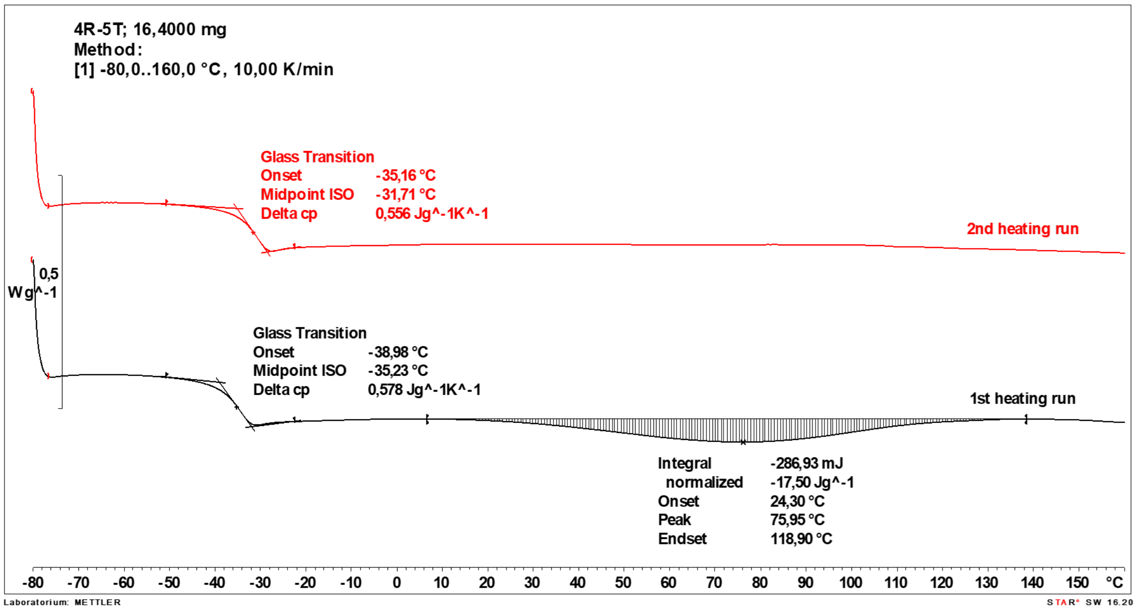


***SI-Figure S17 – multi-page:*** *DSC trace of (e) 0R-5T (polyMEA with 5 wt.% of nano-silica); (f) 4R-5T (polyMEA with 4 wt.% of clay and 5 wt.% of nano-silica).*

***SI-Figure S17 – multi-page (continued, last image):***

**4R-15T**

(g)

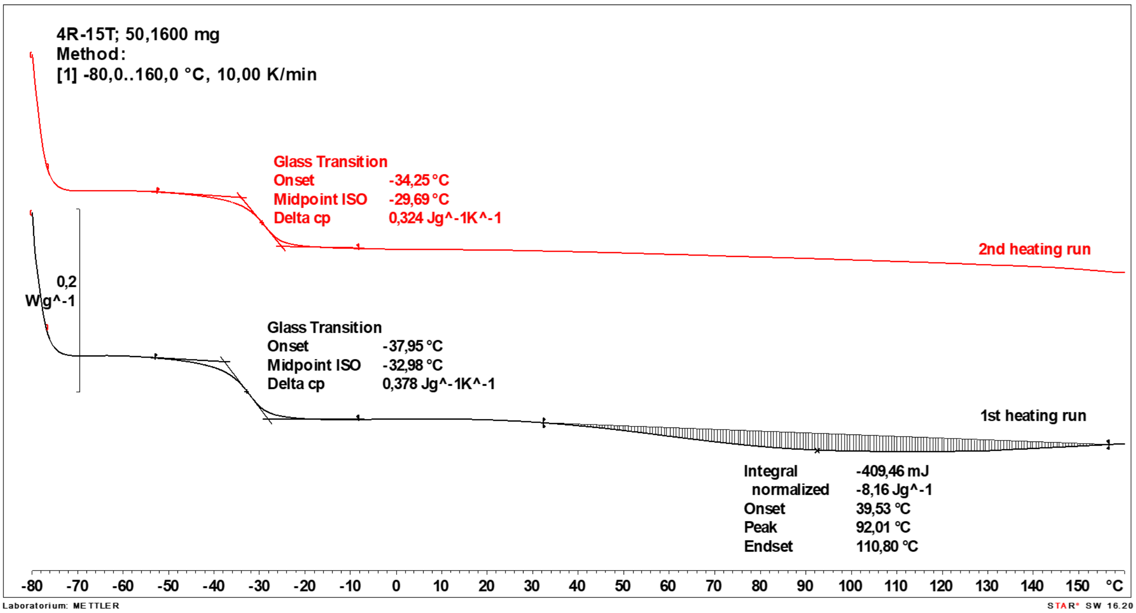


***SI-Figure S17: – multi-page (continued, last image):*** *(g) DSC trace of 4R-15T (polyMEA with 4 wt.% of clay and 15 wt.% of nano-silica).*

# 5. Stability against oxidative and thermal degradation (TGA)

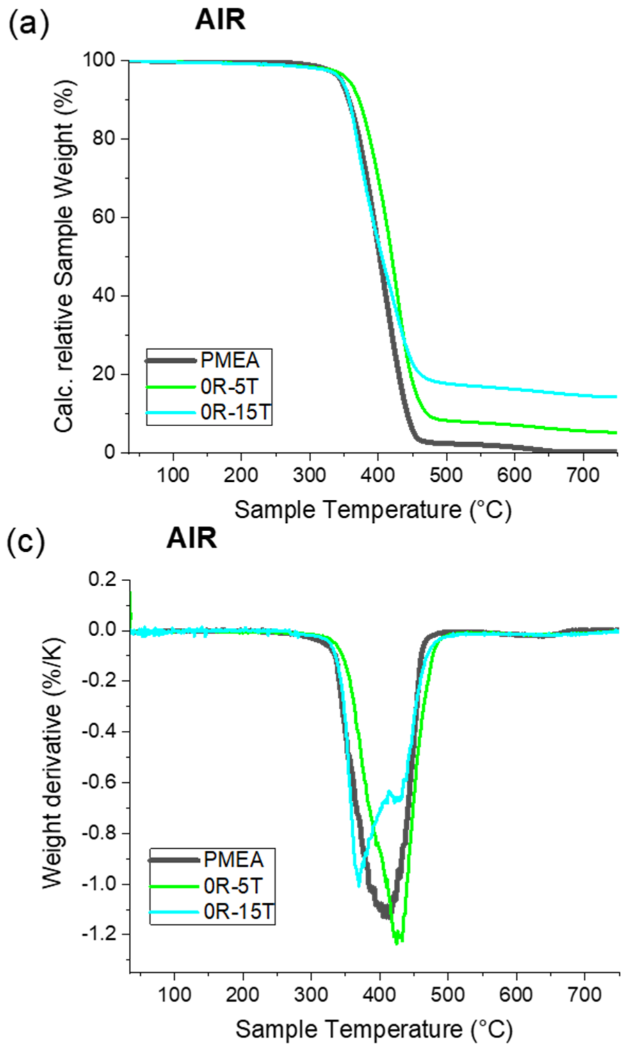


***SI-Figure S18:*** *TGA traces of the doubly filled polyMEA/clay/silica nanocomposite elastomers: Effect of the silica content (0, 5, 15 wt.%) in combination with 4 wt.% of clay: (a, c) in air; (b, d) in nitrogen atmosphere; (a,b) temperature-dependent relative weight; (c, d) derivative of the relative weight (dTG, decomposition peaks).*

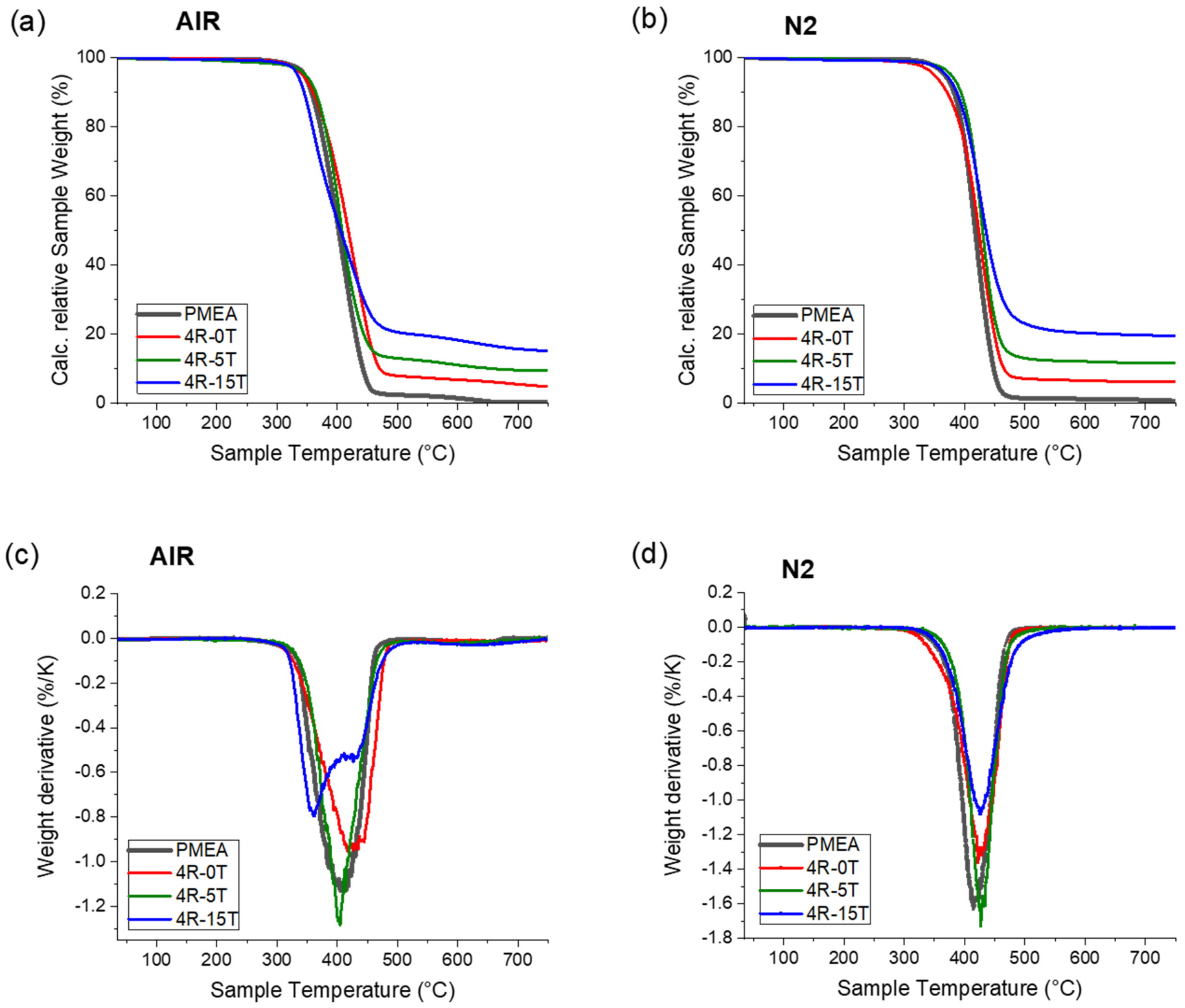


***SI-Figure S19:*** *TGA traces of the polyMEA/clay/silica nanocomposite elastomers: Effect of the silica content (0, 5, 15 wt.%): (a, c) in air; (b, d) in nitrogen atmosphere; (a,b) temperature-dependent relative weight; (c, d) derivative of the relative weight (dTGA, decomposition peaks).*
